# Treatment and Outcome Analysis of 639 Relapsed Non-Hodgkin Lymphomas in Children and Adolescents and Resulting Treatment Recommendations

**DOI:** 10.3390/cancers13092075

**Published:** 2021-04-25

**Authors:** Birgit Burkhardt, Mary Taj, Nathalie Garnier, Veronique Minard-Colin, Volkan Hazar, Karin Mellgren, Tomoo Osumi, Alina Fedorova, Natalia Myakova, Jaime Verdu-Amoros, Mara Andres, Edita Kabickova, Andishe Attarbaschi, Alan Kwok Shing Chiang, Eva Bubanska, Svetlana Donska, Lisa Lyngsie Hjalgrim, Jacek Wachowiak, Anna Pieczonka, Anne Uyttebroeck, Jelena Lazic, Jan Loeffen, Jochen Buechner, Felix Niggli, Monika Csoka, Gergely Krivan, Julia Palma, G. A. Amos Burke, Auke Beishuizen, Kristin Koeppen, Stephanie Mueller, Heidi Herbrueggen, Wilhelm Woessmann, Martin Zimmermann, Adriana Balduzzi, Marta Pillon

**Affiliations:** 1 Department of Pediatric Hematology, Oncology and BMT, University Hospital Muenster, 48149 Münster, Germany; kristin.koeppen@ukmuenster.de (K.K.); Stephanie.mueller@ukmuenster.de (S.M.); heidrun.herbrueggen@charite.de (H.H.); 2 Department of Pediatric Oncology, Royal Marsden Hospital, Surrey SM2 5PT, UK; Mary.Taj@icr.ac.uk; 3 Hospices Civils de Lyon, Institute of Pediatric Hematology and Oncology, 69002 Lyon, France; Nathalie.GARNIER@ihope.fr; 4 Department of Pediatric and Adolescent Oncology, Gustave Roussy, Université Paris-Saclay, 94805 Villejuif, France; Veronique.MINARD@gustaveroussy.fr; 5 Memorial Healthcare Group Private Antalya Yildiz Hospital, Pediatric Oncology Unit, Antalya 07070, Turkey; hazar@akdeniz.edu.tr; 6 Department of Pediatric Oncology and Hematology, Sahlgrenska University Hospital, The Queen Silvia Children’s Hospital, 41685 Gothenburg, Sweden; karin.mellgren@vgregion.se; 7 Children’s Cancer Centre, National Centre for Child Health and Development, Tokyo 104-0045, Japan; osumi-t@ncchd.go.jp; 8 Belarusian Research Centre for Pediatric Oncology, Hematology and Immunology, Research Department, 223053 Minsk, Belarus; a.fedorova@kinderklinik-datteln.de; 9 National Research Centre for Pediatric Hematology, Oncology and Immunology, 129090 Moscow, Russia; nmiakova@mail.ru; 10 Department of Pediatric Hematology and Oncology, University Hospital Valencia, 46010 Valencia, Spain; verdu_josamo@gva.es; 11 Department of Pediatric Oncology, University Hospital Le Fe, 46026 Valencia, Spain; andres_mdm@gva.es; 12 Department of Pediatric Hematology and Oncology, Charles University & University Hospital Motol, 150 06 Prague, Czech Republic; Edita.Kabickova@fnmotol.cz; 13 St. Anna Children’s Hospital, Department of Paediatric Haematology and Oncology, Medical University of Vienna, 1090 Vienna, Austria; andishe.attarbaschi@stanna.at; 14 Department of Pediatrics & Adolescent Medicine, Li Ka Shing Faculty of Medicine, The University of Hong Kong, Queen Mary Hospital, Hong Kong; chiangak@hku.hk; 15 Clinic of Pediatric Oncology and Hematology, Slovak Health University and Children’s Faculty Hospital, 97409 Banská Bystrica, Slovakia; ebubanska@dfnbb.sk; 16 Centre of Pediatric OncoHematology and BMT, National Ukrainian Children’s Hospital Ochmatdyt, 04070 Kiev, Ukraine; donska_s@voliacable.com; 17 Department of Pediatrics and Adolescent Medicine, 2100 Copenhagen, Denmark; lisa.lyngsie.hjalgrim@regionh.dk; 18 Department of Pediatric Oncology, Hematology and Transplantology, Poznan University of Medical Sciences, 60572 Poznan, Poland; jacek.wachowiak@ump.edu.pl (J.W.); annapieczonka@ump.edu.pl (A.P.); 19 Department of Pediatric Hematology and Oncology, University Hospital Leuven, KU Leuven, 3000 Leuven, Belgium; anne.uyttebroeck@uzleuven.be; 20 Department for Hematology and Oncology, University Children’s Hospital, School of Medicine, University of Belgrade, 11000 Belgrade, Serbia; jelena.lazic@udk.bg.ac.rs; 21 Princess Máxima Centre for Pediatric Oncology, 3584 CS Utrecht, The Netherlands; J.L.C.Loeffen@prinsesmaximacentrum.nl (J.L.); A.Beishuizen-2@prinsesmaximacentrum.nl (A.B.); 22 Department of Pediatric Hematology and Oncology, Oslo University Hospital, 0424 Oslo, Norway; jocbuc@ous-hf.no; 23 Department of Pediatric Oncology, University Children’s Hospital Zurich, 8032 Zürich, Switzerland; felix.niggli@kispi.uzh.ch; 24 2nd Department of Pediatrics, Semmelweis University, 1085 Budapest, Hungary; csoka.monika@med.semmelweis-univ.hu; 25 Central Hospital of Southern Pest—National Institute of Hematology and Infectious Diseases, Department for Pediatric Hematology and Hemopoietic Stem Cell Transplantation, 1097 Budapest, Hungary; krivang@hu.inter.net; 26 Faculty of Medicine, Hospital Luis Calvo Mackenna, Department of Pediatrics—Bone Marrow Transplantation Unit, University of Chile, Santiago 8820808, Chile; jpalmab@vtr.net; 27 Department of Pediatric Haematology and Oncology and Palliative Care, Addenbrooke’s Hospital, Cambridge University Hospitals NHS Foundation Trust, Cambridge CB2 0QQ, UK; amos.burke@addenbrookes.nhs.uk; 28 Pediatric Hematology and Oncology, University Medical Centre Hamburg-Eppendorf (UKE), 20246 Hamburg, Germany; w.woessmann@uke.de; 29 Hannover Medical School, Department of Pediatric Hematology and Oncology, 30625 Hannover, Germany; Zimmermann.Martin@mh-hannover.de; 30 Pediatric Hematology and Oncology, Clinica Pediatrica Università degli Studi di Milano Bicocca, Fondazione MBBM, Ospedale San Gerardo, 20126 Monza, Italy; abalduzzi@fondazionembbm.it; 31 Department of Child and Woman Health, Oncology Hematology Division, University-Hospital of Padua, 35125 Padua, Italy; marta.pillon@unipd.it

**Keywords:** refractory and relapsed non-Hodgkin lymphoma, children and adolescents, stem cell transplant

## Abstract

**Simple Summary:**

Despite very poor survival, controversies remain in the treatment for refractory or relapsed non-Hodgkin lymphoma (r/r NHL) in children and adolescents. The current project identifies and reports international experience on re-induction treatment of r/r NHL, hematopoietic stem cell transplantation, risk factors associated with outcome, and suggests treatment recommendations.

**Abstract:**

Despite poor survival, controversies remain in the treatment for refractory or relapsed pediatric non-Hodgkin lymphoma (r/r NHL). The current project aimed to collect international experience on the re-induction treatment of r/r NHL, hematopoietic stem cell transplantation (HSCT), risk factors associated with outcome, and to suggest treatment recommendations. Inclusion criteria were (i) refractory disease, disease progression or relapse of any NHL subtype except anaplastic large cell lymphoma, (ii) age < 18 years at initial diagnosis, (iii) diagnosis in/after January 2000. Data from 639 eligible patients were evaluable. The eight-year probability of overall survival was 34 ± 2% with highly significant differences according to NHL subtypes: 28 ± 3% for 254 Burkitt lymphoma/leukemia, 50 ± 6% for 98 diffuse large B-cell lymphomas, 57 ± 8% for 41 primary mediastinal large B-cell lymphomas, 27 ± 3% for 177 T-lymphoblastic lymphomas, 52 ± 10% for 34 precursor-B-cell lymphoblastic lymphomas and 30 ± 9% for 35 patients with rare NHL subtypes. Subtype-specific factors associated with survival and treatment recommendations are suggested. There were no survivors without HSCT, except in few very small subgroups. Conclusions: There is an urgent need to further improve survival in r/r NHL. The current study provides the largest real-world series, which underlines the role of HSCT and suggests treatment recommendations.

## 1. Introduction

Non-Hodgkin lymphoma (NHL) is the fourth most common type of cancer in children and adolescents. Clinical registries and international cooperative clinical trials led to significant increases in event-free survival [1]. A major step forward was recognizing that different histological NHL subgroups require different treatment approaches [2]. In contrast to leukemia, almost no patient in first complete remission (CR1) qualifies for high-dose (HD) treatment and hematopoietic stem cell transplant (HSCT), either autologous or allogeneic [3]. High-dose treatment and HSCT are reserved for refractory, progressive or relapsed cases. For autologous HSCT, peripheral blood stem cells (PBSC) or bone marrow (BM) are harvested during first- or second-line therapy and cryopreserved for the individual patient. After myeloablative high-dose treatment, the patient is rescued with the harvested cells. For an allogeneic HSCT, PBSC or BM is harvested from another individual. In addition to the cytostatic effect of the high-dose treatment, the substitution of the patient’s immune system by the immune system of the donor results in the graft versus lymphoma effect after allogeneic HSCT.

NHL relapse rates vary according to histological subtype from approximately 25% for anaplastic large cell lymphoma [4], 10–15% for lymphoblastic lymphoma (LBL) [5,6,7,8,9,10,11] and 4–10% for B-NHL [3,8,12,13,14,15,16,17,18,19,20,21,22,23,24,25,26]. Data on relapse rates usually cover cases of refractory disease, disease progression and relapse after CR1 (r/r NHL). In rare NHL subtypes like peripheral T-cell lymphoma (PTCL) [27,28], primary mediastinal large B-cell lymphoma [29,30,31] or non-further classified NHL (other NHL), valid data are lacking.

Only two prospective clinical trials are reported for pediatric r/r NHL, both recruiting r/r B-NHL. The first trial evaluated a response and therapy-related toxicities of rituximab plus ifosfamide, carboplatin, and etoposide (ICE) [32]. The trial was stopped early for insufficient accrual after enrollment of 20 patients. The second trial evaluates ibrutinib in combination with chemo-immunotherapy (NCT02703272) [33]. Part one was recently published with 21 patients showing that ibrutinib can be combined with RICE [32] (rituximab plus ICE) or RVICI [20] (rituximab, vincristine, ifosfamide, carboplatin, idarubicin, and dexamethasone). In both trials, the type and details of HSCT were not part of the clinical assessment.

Due to the lack of consistent data on r/r NHL in children and adolescents, the current study aims to analyze international strategies on re-induction treatment and HSCT in r/r NHL. We provide helpful data in the decision-making process for children and adolescents with r/r NHL.

## 2. Materials and Methods

The study was performed within the network of the International Berlin-Frankfurt-Muenster group (I-BFM) and the European Inter-Group for Childhood and Adolescent Non-Hodgkin Lymphoma (EICNHL). Each national group ensured that the transfer of data was covered by the respective ethics committee and data protection rules. Data on eligible cases were collected on a study-specific case report form. Further information is provided in the Supplementary Materials.

For statistical analyses, overall survival (OS) after relapse was calculated from the date of relapse to date of death from any cause or last follow-up. Survival probabilities were estimated using the Kaplan–Meier method, with differences compared using the log-rank test. Associations of the types of HSCT with patients’ characteristics were analyzed using the χ^2^ test. The selection bias of the patient groups undergoing allogeneic HSCT or autologous HSCT did not allow multivariate analysis. Statistical analyses were performed using SAS (version 9.4; SAS Institute, Cary, NC, USA). Data were updated as of October 2018.

## 3. Results

A total of 639 evaluable cases were included in the analysis (Table 1, Figure S1 and Supplementary Results). The median age at initial diagnosis was 10.8 years (ranging from 0.3–17.9), 464 patients (73%) were male. Histological NHL subtypes were 254 Burkitt lymphomas/leukemias (BL/B-AL), 98 diffuse large B-cell lymphomas (DLBCL), 41 primary mediastinal large B-cell lymphomas, 12 not otherwise specified mature B-NHL, 177 T-LBL, 34 precursors B-cell LBL (pB-LBL), 17 peripheral T-cell lymphomas (PTCL) and 6 rare or not further classified NHL. For B-NHL patients, first-line treatment included French-American-British (FAB) treatment regimen [12,13,14], NHL-BFM regimen [15], CHOP or CHOEP, including etoposide [34], DA-R-EPOCH [30] or other, individualized regimens. Patients with LBL were treated with regimens similar to those used for acute lymphoblastic leukemia (ALL), e.g., EURO-LB02-type treatment (97%) [7]. In PTCL, the first-line treatment was an ALL-type regimen in 5, B-NHL courses in 2, CHO(E)P in 2 and individualized regimens in the remaining PTCL patients (Table S1).

**Figure 1:**
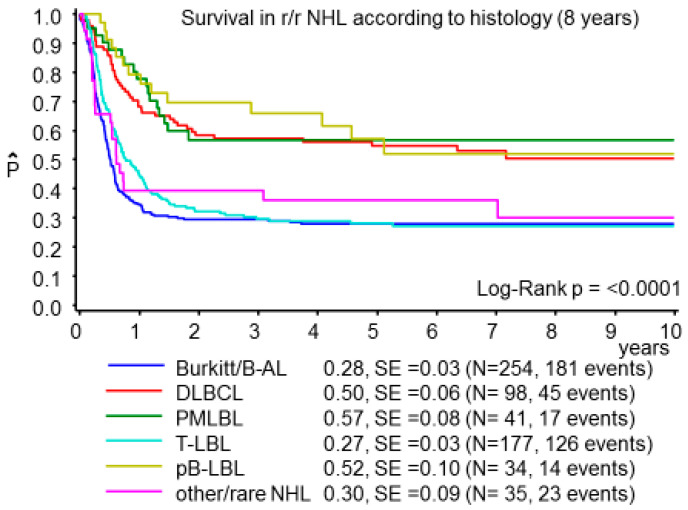
Probability of survival at 8 years for all r/r NHL according to histological subtypes. r/r NHL: refractory, progressive or relapsed non-Hodgkin lymphoma; B-AL: Burkitt leukemia; DLBCL: diffuse large B-cell lymphoma; PMLBL: primary mediastinal large B-cell lymphoma; T-LBL: T-cell lymphoblastic lymphoma; pB-LBL: precursor B-cell lymphoblastic lymphoma.

Overall survival at 8 years for the whole cohort was 34 ± 2% with highly significant differences by histological subtypes with 57 ± 8% for primary mediastinal large B-cell lymphoma, 52 ± 10% for pB-LBL, 50 ± 6% for DLBCL, 32 ± 9% for rare or not further classified NHL subtypes, including B-NHL nos and PTCL, 28 ± 3% for BL/B-AL and 27 ± 3% for T-LBL patients (log-rank *p* < 0.0001, Figure 1). Of the 639 evaluable patients, 23% underwent autologous HSCT, 39% allogeneic HSCT, and 37% did not achieve HSCT with only individual patients alive (Tables S2 and S3 and Figures S2 and S3).

### 3.1. T-Cell Lymphoblastic Lymphoma

**Figure 2:**
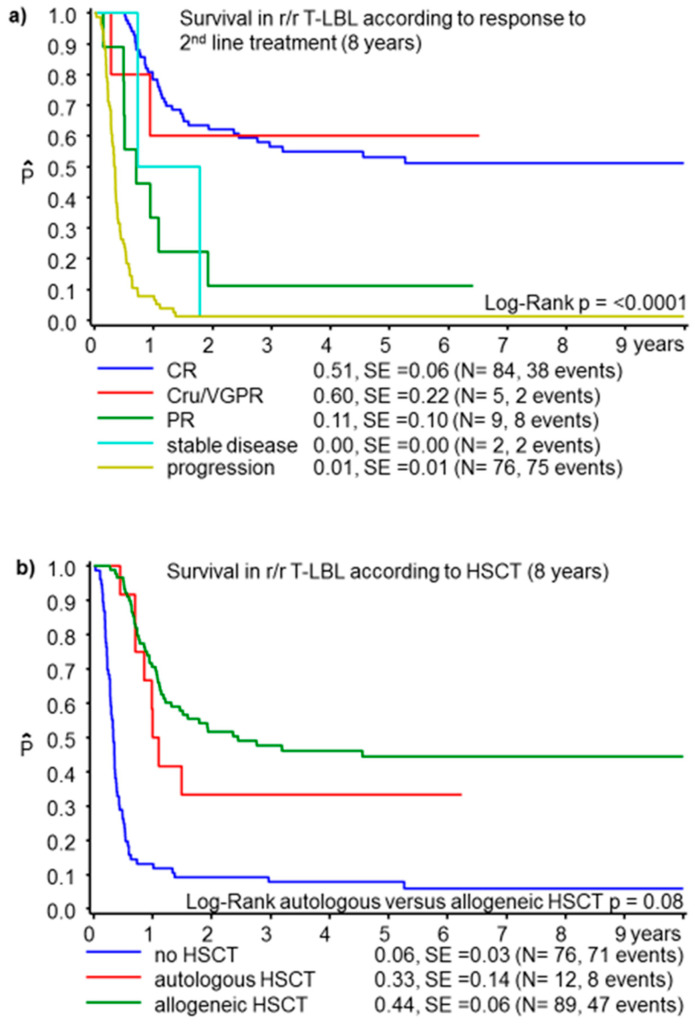
Probability of survival at 8 years for T-cell lymphoblastic lymphoma (T-LBL) according to remission status after second-line treatment (**a**) and according to HSCT status (**b**). HSCT: hematopoietic stem cell transplantation; CR: complete remission; Cru: unconfirmed complete remission; VGPR: very good partial remission; PR: partial remission; SD: stable disease.

The 8-years OS for the 177 r/r T-LBL was 27 ± 3%. Table 2 provides the comparison of T-LBL characteristics and univariate analyses of parameters potentially associated with outcome. Failures occurred in a median of 11 (0.1–82) months from the initial diagnosis. Second-line treatment comprised elements from high-risk ALL or relapsed ALL protocols in most patients (152, 47 alive), while 5 patients received ICE (2 alive) and 4 patients received courses from B-NHL protocols (all died) or other individual regimens (16, 2 alive). Seventy-six r/r T-LBL (43%) did not receive HSCT. The majority of them (69/76) did not reach HSCT due to lymphoma progression and died. HSCT was not planned in 2 patients with refractory disease (both alive) and 5 patients with very late relapses (3 alive, Table S3). A total of 101 (57%) patients received HSCT. Among patients who did not undergo HSCT, there were more patients 10 years or older, fewer patients with central nervous system (CNS) involvement at relapse, more patients with local relapses, early events (3–6 months after primary diagnosis), and disease progression during second-line treatment compared to patients consolidated by HSCT (Table 2). Twelve patients received HD treatment followed by autologous HSCT, and 89 patients underwent allogeneic HSCT. The only significant difference between these cohorts was a higher proportion of patients with CNS involvement at relapse among those receiving autologous HSCT. Eight of the 12 patients with autologous HSCT and 73 of the 89 patients with allogeneic HSCT received total body irradiation (TBI)-based conditioning regimen. For the whole cohort of 177 r/r T-LBL, age 10 years or older, stage III disease at initial diagnosis, relapse not involving CNS, local relapse, relapse 3–9 months from initial diagnosis, poor response to second-line treatment and no HSCT for consolidation were significantly associated with inferior survival (Figure 2 and Figure S4).

### 3.2. Precursor B-Cell Lymphoblastic Lymphoma

The 8-years OS for the 34 r/r pB-LBL was 52 ± 10%. The median age at initial diagnosis was 9.3 (1.3–17.9) years. Twenty patients were male (59%). The initial stages of disease at diagnosis were I, II, III and IV in 3%, 15%, 38% and 44% of the patients with bone marrow (BM) involvement in 38% and CNS disease in 12%. The median time to relapse was 26 (1–70) months with 18/34 r/r pB-LBL cases later than 24 months after initial diagnosis. At relapse, 45% presented with BM involvement, 18% with CNS involvement and 73% with involvement of the initial sites. Like r/r T-LBL, most of the patients (26/34) received second-line therapy based on ALL high-risk or ALL relapse protocols. Nine patients did not receive HSCT, which was not achieved in 2 patients (both died) and not planned in 7 patients (6 alive). Three patients underwent HD treatment followed by autologous HSCT (2 alive), 22 patients achieved allogeneic HSCT (12 alive) with TBI-based conditioning in 16 patients (9 alive) and busulfan or treosulfan based conditioning regimen in 6 patients (3 alive).

### 3.3. Diffuse Large B-Cell Lymphoma

**Figure 3:**
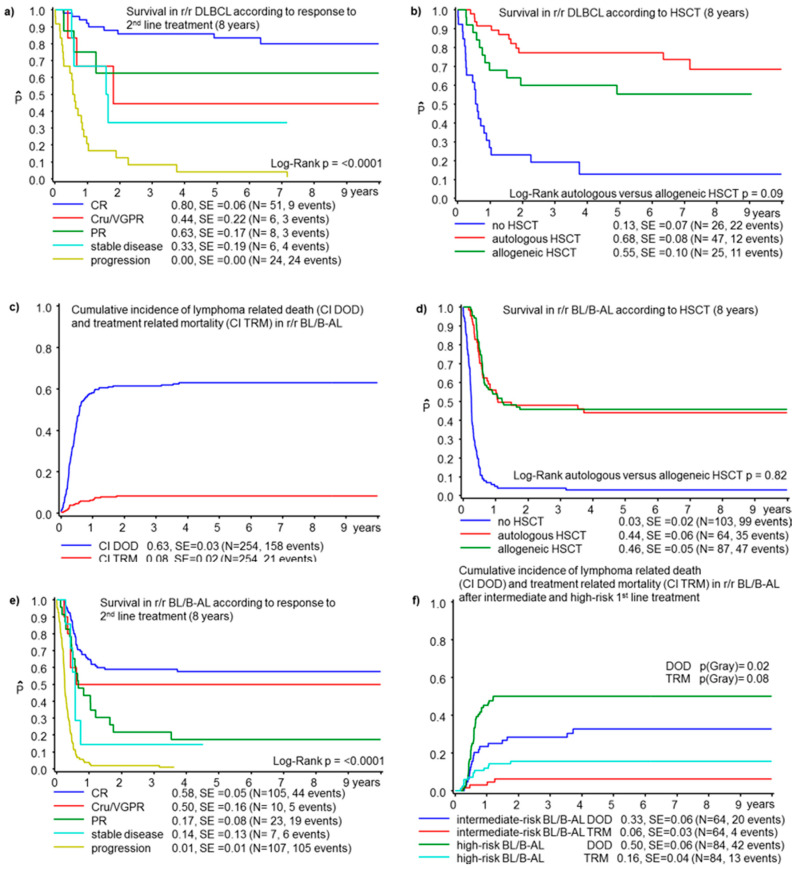
Outcome in mature B-NHL: probability of survival at 8 years for diffuse large B-cell lymphoma (DLBCL) according to remission status after second-line treatment (**a**) and according to HSCT status (**b**). Cumulative incidence of lymphoma related death (CI DOD) and treatment-related mortality (CI TRM) for all r/r Burkitt lymphoma/leukemia (**c**), probability of survival at 8 years according to HSCT status (**d**) and to remission status after second-line treatment (**e**), and cumulative incidence of lymphoma related death and treatment-related mortality for r/r BL/B-AL after intermediate-risk and high-risk first-line treatment (**f**). HSCT: hematopoietic stem cell transplantation; CR: complete remission; Cru: unconfirmed complete remission; VGPR: very good partial remission; PR: partial remission; SD: stable disease; IR: intermediate risk; HR: high-risk.

The 8-years OS for 98 r/r DLBCL was 50 ± 6%. Twenty-four patients suffered r/r DLBCL after FAB-type first-line treatment (17 group B and 7 group C), 67 patients after BFM-type treatment (1 R1, 17 R2, 28 R3, 21 R4), and the remaining 7 patients after DA-EPOCH (1), CHOP (1) or other individual regimens (5). Patient characteristics are detailed in Table 3. The median interval from diagnosis to relapse was 6 (0.4–61) months, with 25% of relapses later than 12 months after diagnosis. The most frequently used second-line regimen was (R)ICE in 34 patients (21 alive), followed by BFM or FAB courses for advanced B-NHL (28, 17 alive), RVICI and variants (9, 4 alive), (R)CHOP/CHOEP (6, 5 alive), or other regimens (21, 6 alive). Twenty-seven percent of r/r DLBCL did not undergo HSCT as it was not planned in 4 patients with late relapses (all alive) and not achieved in 22 patients due to DLBCL progression (all died). The proportion of patients achieving HSCT was higher in the more recent period (Table 3). This may be related to second-line regimens, which were more often (R)ICE or RVICI. Among the 72 patients with HSCT, 25 underwent allogeneic HSCT and 47 autologous HSCT. Characteristics of the patients undergoing allogeneic or autologous HSCT are compared in Table 3. The only significant difference was the sex distribution. The conditioning regimen for autologous HSCT was BEAM (22 patients, 18 alive), busulfan based (15, 9 alive) and individualized regimen (10, 8 alive). For allogeneic HSCT, conditioning regimens were based on TBI (10 patients, 6 alive), busulfan (7, 4 alive) or individualized (8, 4 alive). Variables associated with outcome (Table 3) were response to first-line treatment with inferior OS for patients with refractory disease or progression during treatment, compared with relapses after first-line treatment. Time to relapse, response to second-line treatment and the achievement of HSCT were also found to be significant (Table 3, Figure 3a,b and Figure S5).

### 3.4. Burkitt Lymphoma/Leukemia

The 8-year OS for the 254 r/r patients with BL/B-AL was 28 ± 3%. Sixty-three percent of patients died of lymphoma progression and about 8% from treatment-related mortality, TRM (Figure 3c). First-line treatment had followed current FAB or NHL-BFM protocols in most patients (Table S4). The median interval to failure was 5 (0.4–73) months, including 16 cases (6%) with relapses later than 1 year and 3 patients (1%) later than 3 years after initial diagnosis.

#### 3.4.1. Second-Line Treatment in r/r BL/B-AL

Second-line treatment was initiated with NHL-BFM or FAB courses for advanced B-NHL in 94 patients (30% alive), (R)ICE in 89 patients (29% alive), RVICI plus variants in 33 patients (42% alive), DA-R-EPOCH in 4 patients (all died) and other individualized regimens in 34 patients (15% alive) (Table S5). Patients with advanced disease and intense first-line treatment at initial diagnosis were more likely to receive (R)ICE or RVICI at relapse.

Of the 89 patients, who started second-line treatment with (R)ICE, 60 patients achieved HSCT with OS of 46 ± 10% for 26 patients with autologous and 35 ± 8% for 34 patients with allogeneic HSCT.

Among 33 patients with RVICI re-induction, 13 patients did not achieve HSCT (all died). Two patients underwent autologous HSCT (1 alive), while 18 patients received allogeneic HSCT after RVICI re-induction resulting in OS of 72 ± 10% (Figure S6a,b).

#### 3.4.2. HSCT in BL/B-AL

High-dose chemotherapy with HSCT was planned for 98% of patients with r/r BL/B-AL, but 38% did not achieve HSCT, predominantly because of lymphoma progression (93 lymphoma-associated deaths, 4 TRM). Bone marrow relapses, local relapses, early events and poor response to second-line treatment were significantly more frequent among patients not achieving HSCT, while CNS disease at relapse was less frequent (Table 4). Overall, survival for 103 patients without HSCT was 3 ± 2%, compared with 44 ± 6% for 64 patients with autologous HSCT and 46 ± 5% for 87 patients with allogeneic HSCT (Figure 3d). The incidence of failure and TRM in patients with autologous HSCT were 47 ± 6% and 8 ± 3%, respectively, compared with 39 ± 5% and 14 ± 4% for patients with allogeneic HSCT (Figure S6c,d). The cohorts of patients with autologous and allogeneic HSCT showed significant differences. Allogeneic HSCT was more often applied in the more recent years, while autologous HSCT was more frequently used in the earlier study period. In the allogeneic HSCT cohort, the proportion of female patients was higher. Patients had significantly more advanced disease at initial diagnosis and/or BM involvement at initial diagnosis and/or at relapse (Table 4 and Table S6). The conditioning regimen for autologous HSCT was based on busulfan (25 patients, 10 alive), BEAM [3] (21, 11 alive), TBI (7, 3 alive) or individualized regimens (11, 5 alive). For allogeneic HSCT, the most frequently used conditioning regimens were TBI-based (36 patients, 15 alive), Burkitt-specific combination of rituximab, fludarabine, thiotepa, carboplatin, mitoxantrone, paclitaxel [20] (20, 14 alive), busulfan (13, 5 alive), treosulfan (6, 1 alive) or individualized regimens (12, 5 alive).

#### 3.4.3. Variables Associated with Survival in r/r BL/B-AL

Poor response to first and/or second-line treatment and early events were significantly associated with inferior survival (Table 4, Figure 3e and Figure S6f,g). There was a trend towards an inferior outcome for r/r BL/B-AL with CNS involvement at initial diagnosis (Figure S6e) and BM involvement either at initial diagnosis or at relapse. Involvement of CNS at relapse was not significantly associated with survival; neither for the whole cohort of r/r BL/B-AL nor for patients with autologous or allogeneic HSCT. The interval between initial diagnosis and the diagnosis of r/r Burkitt was highly significantly associated with survival (Figure S6g). Similarly, the intensity of first-line treatment was significantly associated with outcome. Five patients suffered a relapse after first-line treatment for low-risk lymphoma (FAB group A or NHL-BFM group R1), of whom 4 patients are alive, including 2 patients without HSCT. For 95 patients with r/r Burkitt after intermediate-risk B-NHL treatment (FAB group B or NHL-BFM R2 or R3), OS was 39 ± 5%. For r/r BL/B-AL after intense first-line treatment for high-risk patients (FAB group C or NHL-BFM R4), OS was 20 ± 3%. The most relevant cause of death in these patients was lymphoma progression (Figure 3f).

### 3.5. Rituximab in First-Line Treatment of Mature B-NHL

Rituximab in first-line treatment was reported for a total of 30 r/r BL/B-AL patients. Of those, 5 patients were alive (17%), 1 after autologous and 4 after allogeneic HSCT. This is inferior to a survival rate of 31% (68/219) for r/r BL/B-AL without rituximab in first-line treatment. In DLBCL, 36% (5/14) patients with rituximab in first-line therapy are alive, compared with 57% (47/83) in r/r DLBCL without rituximab.

## 4. Discussion

Current risk-adapted first-line protocols for NHL result in event-free survival rates of more than 80% or even 90% [1]. In contrast, survival for patients who suffer relapse is poor (Table S7) [6,9,19,20]. Therefore, there is a clear medical need for improving outcomes [35]. The current manuscript presents by far the largest series of pediatric and adolescent patients with r/r NHL and highlights the importance of international collaboration. These real-world data were contributed by many national groups, which is the great and unique strength of the project. The data will serve as a baseline for new international trials and decision-making processes and are more useful than retrospective series from single groups.

The current data underline the role of HSCT for consolidation in r/r NHL. Survival was in the range of 50% for patients who underwent HSCT, while survival for r/r NHL without HSCT was below 10%. There were only a few low-risk individual patients alive without HSCT. In patients with very late relapses, available molecular techniques support the importance of differentiating late relapses from second malignancies in the future. Despite these rare cases, our study leads to the conclusion that all pediatric patients with r/r NHL have a clear indication for HSCT. Unfortunately, the survival of patients who do not respond to second-line therapy is very poor and short-lived. The key aim of the management for these patients is to give palliative care to improve their quality of life. In individual patients, systemic chemotherapy with limited toxicity may delay or slow down disease progression and contribute to pain control. Some patients may also be offered early phase clinical trials with new compounds, which also may slow down fatal disease progression. Next-generation sequencing (NGS) panels may also identify molecular targets and novel agents that are more suitable for a particular patient.

Patients at risk for poor outcomes were more likely to receive allogeneic HSCT. In addition, differences in the availability of allogeneic HSCT, national guidelines for the treatment of r/r NHL and individual decisions of the treating physicians introduced a selection bias into the current cohort. Therefore, a direct comparison of the survival rates for autologous versus allogeneic HSCT is not possible. Only prospective randomized clinical trials would be able to answer the question on the role of autologous versus allogeneic HSCT in r/r NHL. It is questionable whether such trials would be ethical or feasible. Therefore, the current analyses of this large cohort from multiple countries serve as the most robust evidence base for future treatment decisions in r/r NHL and make it possible to suggest the following treatment recommendations for r/r NHL (summarized in Table S8).

### 4.1. Lymphoblastic Lymphoma

In contrast to other NHL subtypes, patients with T-LBL stage III disease at initial diagnosis did worse than those with stage IV disease [36], and CNS disease was not associated with inferior survival. Time to relapse, response to second-line treatment and achievement of HSCT were the most relevant parameters for survival. In the light of the current results and the available published data [5,6,8,10], the following treatment recommendations are summarized: second-line treatment should comprise intense treatment courses analogous to high-risk ALL or relapsed ALL protocols followed by allogeneic HSCT. In T-LBL, TBI-based conditioning is advised similar to pediatric T-ALL, while in pB-LBL the available data do not allow a clear preference [37]. The final results of the trial ALL SCTped 2012 FORUM showed the superiority of TBI-based conditioning in ALL [38]. Disease progression during second-line treatment is the most frequent cause of death. Therefore, high treatment intensity is needed to achieve remission, which is crucial before HSCT. Management guidelines for r/r LBL, especially for T-LBL, include preventing any treatment delay and early start of subsequent courses of treatment as soon as the patient is clinically stable, relatively independent from hematological recovery. To modulate the immune system after allogeneic HSCT, limited immune suppression and early tapering of immunosuppression after transplant is recommended. These recommendations apply to all r/r LBL with possible exceptions for rare cases with the very early refractory disease with good response to intensified treatment and rare cases with very late relapses of (pB)-LBL that may not require allogeneic HSCT. Interestingly, the survival of pB-LBL in the current analysis is superior to that reported for smaller series in the literature [6,9,10].

As in relapsed ALL, autologous HSCT does not have a major role in the treatment of r/r LBL patients [5,8,39]. The current analyses did identify a subgroup of r/r LBL in whom HD treatment followed by autologous HSCT may be recommended. Given the close biological relationship, progress in the treatment of r/r ALL may translate into new clinical trials for LBL in the future.

### 4.2. Diffuse Large B-Cell Lymphoma

For r/r DLBCL, second-line treatment with (R)ICE became more common in the more recent period. This regimen leads to sufficient response allowing consolidation by HSCT in most patients. The standard of care for most r/r DLBCL is, therefore, RICE re-induction followed by autologous HSCT. Cases with aggressive refractory disease or early progression may require more intensive treatment approaches like those for r/r Burkitt lymphoma. In rare individual cases with very late relapses, limited first-line treatment and favorable response to second-line therapy, HSCT may not be required for consolidation.

### 4.3. Burkitt Lymphoma/Leukemia

Response to second-line treatment is strongly associated with survival in r/r BL/B-AL. The patients who experience disease progression during re-induction die, while almost 60% of patients who achieve a second CR survive. Salvage chemotherapy with courses for high-risk B-NHL as recommended earlier [22,40] have become outdated as more effective re-induction regimens like RICE and RVICI became available [20,32,33]. In the current analyses, survival was similar for r/r BL/B-AL with autologous and allogeneic HSCT. However, high-risk r/r BL/B-AL were overrepresented in the cohort of patients consolidated by allogeneic HSCT, so a recommendation in favor of one or the other type of HSCT cannot be made. Instead, the decision for autologous or allogeneic HSCT in patients with r/r BL/B-AL needs to be made on an individual patient basis taking into account the medical condition, the availability of autologous cells or allogeneic grafts, the experience of the treating institution, and the timing of relapse. Data on graft versus lymphoma effect in Burkitt lymphoma are limited and do not support allogeneic over autologous HSCT per se in this disease [41,42,43,44,45]. It has been shown that each progression is associated with additional chemo-resistance caused by increasing genetic alterations [46]. Therefore, the aim of the second-line treatment is to avoid progressions by maintaining a high time- and dose-intense treatment and achieve CR before HSCT. This can make it difficult to harvest autologous stem cells, which by definition requires full hematological recovery and often additional days for arranging the harvest. As the incidence of lymphoma-associated deaths by far exceeds treatment-related deaths, the harvest of autologous stem cells during re-induction with minimal risk of disease progression remains challenging. For patients, who have cryopreserved autologous cells available from first-line therapy, both autologous and allogeneic HSCT is feasible, and the decision may be based on the individual risk profile of the patient. For patients without cryopreserved autologous cells available at the time of relapse, it remains the responsibility of the treating physicians to balance the two goals of high treatment intensity with allogeneic HSCT (without delays for stem cell harvest) versus lower treatment-related mortality favoring autologous HSCT.

Current still limited clinical experiences reported that the outcome of r/r BL/B-AL in children and adolescents is poor for patients who received rituximab as part of first-line treatment. Interestingly, although survival of patients with r/r BL/B-AL with rituximab in first-line was inferior to those of rituximab naïve r/r BL/B-AL, a few surviving patients were reported. Given the above-mentioned time selection bias, additional data are needed. Rescuing these patients will be challenging in the future.

## 5. Conclusions

The current data will form the basis of treatment selection for patients and planning future clinical trials in r/r NHL. Further intensification of re-induction treatment (with either increased dose or addition of other agents) with cytostatic agents cannot solve the problem of disease resistance at relapse. To overcome this resistance, molecular profiling of the disease and drugs with new mechanisms of action is required. For B-cell malignancies, several new drugs fulfilling this need are available [35]. In the database of ClinicalTrials.gov (accessed on 4 March 2021), roughly 70 interventional trials are open for recruitment of refractory or relapsed NHL (Table S9). The vast majority of the trials are not limited to NHL but open for various diagnoses. The impact of these (basket) trials on the outcome for r/r NHL will be limited as the trial designs usually do not address the specific medical needs of patients with highly aggressive and fast proliferating NHL. However, new approaches with CAR T-cell products, bispecific antibodies, or antibody–drug conjugates adequately adapted to NHL disease kinetics may pave the way for increasing remission rates before HSCT and higher survival rates in consequence. In parallel, increasing knowledge on NHL biology and molecular genetic characterization has already contributed to identifying genetically defined subgroups [47,48,49,50,51,52,53,54,55]. The era of targeted therapy, small molecules and gene editing will provide new perspectives for patients with refractory or relapsed NHL. Smartly coordinated international efforts are needed to design and conduct clinical trials that make these new compounds available for pediatric patients with r/r NHL. The primary aim of these trials is to improve survival rates, which is only achievable by structured clinical trials. The data presented will be an invaluable asset in planning these future trials. In summary, the current pooled international data provides comprehensive treatment and outcome information that will support systematic trials and the everyday decision-making processes for r/rNHL patients in the future.

## Figures and Tables

**Table 1:** Patients’ characteristics and association with outcome in r/r NHL patients. All data refer to cases with respective information available. OS 8 y: probability of overall survival 8 years from relapse; SE: standard error; BM: bone marrow; CNS: central nervous system; CR: complete remission; Cru: unconfirmed CR; VGPR: very good partial remission; PR: partial remission; SD: stable disease; HSCT: hematopoietic stem cell transplantation.

Patients’ Characteristics and Association with Outcome	*n*	OS 8 y (±SE) (%)	*p* Value (Log-Rank)
Sex			
Male	464	34 ± 2	
Female	175	34 ± 4	0.70
Age			
<10 years	287	38 ± 3	
>10 years	352	31 ± 3	0.084
Period of diagnosis			
2000–07	324	34 ± 3	
2008–16	315	34 ± 3	0.65
Initial stage of disease			
I	12	53 ± 16	
II	34	68 ± 8	
III	361	34 ± 3	
IV	218	28 ± 3	0.0003
BM involvement at initial diagnosis			
Yes	191	25 ± 3	
No	435	38 ± 3	0.0003
BM involvement at relapse			
Yes	198	28 ± 3	
No	435	36 ± 3	0.0002
CNS involvement at initial diagnosis			
Yes	76	27 ± 5	
No	550	36 ± 2	0.011
CNS involvement at relapse			
Yes	128	32 ± 4	
No	505	34 ± 2	0.76
Local relapse			
Yes	527	33 ± 2	
No	103	37 ± 5	0.34
Response to first-line treatment			
Refractory	43	30 ± 7	
Progression	118	28 ± 4	
Relapse	478	36 ± 2	0.001
Time to r/r disease			
<3 months from the initial diagnosis	76	25 ± 5	
3–6 months	232	27 ± 3	
6–9 months	104	34 ± 5	
>9 months	217	45 ± 4	<0.0001
Response to second-line treatment			
2nd CR	294	61 ± 3	
CRu or VGPR	29	44 ± 12	
PR	54	34 ± 7	
SD	18	27 ± 11	
Progression	238	1 ± 1	<0.0001
HSCT			
No HSCT	238	8 ± 2	
Autologous HSCT	150	55 ± 5	
Allogeneic HSCT	251	47 ± 3	<0.0001

**Table 2:** Association of patients’ characteristics and response parameters with outcome in r/r T-LBL patients and detailed comparison of r/r T-LBL patients treated without HSCT (no HSCT), with autologous (auto) HSCT and with allogeneic (allo) HSCT. All data refer to patients for whom the relevant variable was known.

Patients’ Characteristics		No	pOS 8 y (±SE)	*p* Value (Log-Rank)	No HSCT *n* = 76	Autologous HSCT *n* = 12	Allogeneic HSCT *n* = 89	*p* Value No HSCT vs. HSCT (Auto or Allo) (chi^2^)	*p* Value Auto vs. Allo (chi^2^)
		*n* = 177	(%)		(%)	(%)	(%)		
Diagnosis	2000–07	84	27 ± 5		46	58	47		
	2008–16	93	27 ± 5	0.82	54	42	53	0.7454	0.4685
Sex	Male	137	28 ± 4		80	63	74		
	Female	40	24 ± 7	0.64	20	17	26	0.4297	0.4893
Age	<10 years	83	35 ± 5		38	42	55		
	≥10 years	94	20 ± 4	0.0032	62	58	45	0.0434	0.3827
Stage	I	0			0	0	0		
	II	4	50 ± 25		1	0	4		
	III	119	21 ± 4		82	92	61		
	IV	42	38 ± 8	0.019	17	8	35	0.0580	0.1133
Initial CNS disease	Yes	11	51 ± 16		4	8	8		
	No	155	26 ± 4	0.19	96	92	92	0.2823	0.9907
Initial BM disease	Yes	35	33 ± 8		16	8	28		
	No	131	24 ± 4	0.11	84	92	72	0.1268	0.1488
CNS disease at relapse	Yes	33	45 ± 9		9	50	23		
	No	144	24 ± 4	0.14	91	50	77	0.0052	0.0406
BM disease at relapse	Yes	62	27 ± 6		38	8	36		
	No	115	27 ± 4	0.41	62	92	64	0.4490	0.0555
Local relapse	Yes	144	22 ± 4		90	67	76		
	No	33	49 ± 9	0.003	10	33	24	0.0162	0.4631
Response to first-line treatment	Refractory	9	22 ± 14		9	0	2		
	Progression	19	37 ± 11		15	17	7		
	Relapse	149	26 ± 4	0.46	76	84	91	0.0271	0.4374
Time to relapse	<3 months	17	35 ± 12		12	9	8		
	3–6 months	30	7 ± 5		26	18	10		
	6–9 months	20	12 ± 8		15	9	9		
	≥9 months	107	35 ± 5	<0.0001	47	64	73	0.0091	0.8689
Response to second-line treatment	CR	84	51 ± 6		9	91	75		
	Cru/VGPR	5	60 ± 22		0	0	6		
	PR	9	11 ± 10		3	0	8		
	SD	2	0		0	0	2		
	PD	76	1 ± 1	<0.0001	88	9	9	<0.0001	0.7282
HSCT	No	76	6 ± 3						
	Autologous	12	33 ± 13						
	Allogeneic	89	44 ± 6	<0.0001					

**Table 3:** Association of patient’s characteristics and response parameters with outcome in r/r DLBCL patients and detailed comparison of r/r DLBCL patients treated without HSCT (no HSCT), with autologous (auto) HSCT and with allogeneic (allo) HSCT. All data refer to patients for whom the relevant variable was known.

Patients’ Characteristics		No	pOS 8 y (±SE)	*p* Value (Log-Rank)	No HSCT *n* = 26	Autologous HSCT *n* =47	Allogeneic HSCT *n* = 25	*p* Value No HSCT vs. HSCT (Auto or Allo) (chi^2^)	*p* Value Auto vs. Allo (chi^2^)
		*n* = 98	(%)		(%)	(%)	(%)		
Diagnosis	2000–07	57	51 ± 7		77	55	44		
	2008–16	41	52 ± 8	0.93	23	44	56	0.0237	0.3603
Sex	Male	60	58 ± 7		62	70	44		
	Female	38	40 ± 8	0.12	39	30	56	0.9694	0.0298
Age	<10 years	39	55 ± 9		42	40	36		
	>10 years	59	47 ± 7	0.45	58	69	64	0.7602	0.7138
Stage	I	4	75 ± 22		4	6	0		
	II	10	80 ± 13		4	17	4		
	III	64	44 ± 7		72	60	72		
	IV	19	52 ± 12	0.32	20	17	24	0.6847	0.2025
Initial CNS disease	Yes	10	48 ± 16		8	6	20		
	No	87	51 ± 6	0.50	92	94	80	0.6594	0.0801
Initial BM disease	Yes	11	64 ± 15		12	13	8		
	No	86	49 ± 6	0.68	88	87	92	0.9703	0.5763
CNS disease at relapse	Yes	13	42 ± 15		12	11	20		
	No	83	50 ± 6	0.47	88	89	80	0.7267	0.3085
BM disease at relapse	Yes	12	67 ± 14		12	11	16		
	No	84	47 ± 6	0.41	88	89	84	0.8622	0.5582
Local relapse	Yes	89	51 ± 6		96	98	88		
	No	5	40 ± 22	0.44	4	2	12	0.7316	0.0819
Response to first-line treatment	Refractory	9	33 ± 16		8	9	12		
	Progression	22	35 ± 10		31	17	24		
	Relapse	67	58 ± 7	0.016	62	75	64	0.4920	0.6487
Time to relapse	<3 months	12	25 ± 13		23	6	13		
	3–6 months	29	44 ± 11		27	28	38		
	6–9 months	20	43 ± 13		15	28	13		
	>9 months	36	70 ± 8	0.011	35	38	38	0.2694	0.4198
Response to second-line treatment	CR	51	80 ± 6		16	59	83		
	Cru/VGPR	6	44 ± 22		0	11	4		
	PR	8	63 ± 17		0	13	8		
	SD	6	33 ± 19		0	13	0		
	PD	24	0	<0.0001	84	4	4	<0.0001	0.2235
HSCT	No	26	13 ± 7						
	Autologous	47	68 ± 8						
	Allogeneic	25	55 ± 10	<0.0001					

**Table 4:** Association of patient’s characteristics and response parameters with outcome in r/r BL/B-AL patients and detailed comparison of r/r BL/B-AL patients treated without HSCT, with autologous HSCT and with allogeneic HSCT. All data refer to patients for whom the relevant variable was known.

Patients’ Charateristics		No	pOS 8 y (±SE)	*p* Value (Log-Rank)	No HSCT *n* = 103	Autologous HSCT *n* = 64	Allogeneic HSCT *n* = 87	*p* Value No HSCT vs. HSCT (Auto or Allo) (chi^2^)	*p* Value Auto vs. Allo (chi^2^)
		*n* = 254	(%)		(%)	(%)	(%)		
Diagnosis	2000–07	135	26 ± 4		54	64	44		
	2008–16	119	30 ± 4	0.32	46	36	56	0.7477	0.0132
Sex	Male	207	29 ± 3		77	92	79		
	Female	47	26 ± 6	0.30	23	8	21	0.1040	0.0295
Age	<10 years	134	32 ± 4		49	47	62		
	≥10 years	120	24 ± 4	0.18	52	53	38	0.2668	0.0633
Stage	I	3	67 ± 27		1	3	0		
	II	14	57 ± 13		6	6	5		
	III	107	31 ± 5		41	59	31		
	IV	129	22 ± 4	0.15	52	31	64	0.9807	0.0005
Initial CNS disease	Yes	46	13 ± 5		19	11	23		
	No	207	31 ± 3	0.039	81	89	77	0.8800	0.0562
Initial BM disease	Yes	121	20 ± 4		49	30	60		
	No	132	36 ± 4	0.069	51	70	40	0.7548	0.0003
CNS disease at relapse	Yes	67	25 ± 5		20	27	35		
	No	185	29 ± 3	0.74	80	73	65	0.0386	0.3285
BM disease at relapse	Yes	97	25 ± 4		46	21	43		
	No	155	30 ± 4	0.091	54	79	57	0.0413	0.0050
Local relapse	Yes	203	28 ± 3		87	79	75		
	No	48	29 ± 7	0.44	13	21	25	0.0388	0.5061
Response to first-line treatment	Refractory	12	8 ± 8		6	6	2		
	Progression	56	12 ± 4		32	16	15		
	Relapse	186	43 ± 4	<0.0001	62	78	83	0.0037	0.4589
Time to relapse	<3 months	36	17 ± 6		19	11	12		
	3–6 months	142	22 ± 4		62	63	46		
	6–9 months	46	38 ± 7		14	19	24		
	≥9 months	26	57 ± 10	<0.0001	6	8	18	0.0400	0.1770
Response to second-line treatment	CR	105	58 ± 5		5	62	70		
	Cru/VGPR	10	50 ± 16		0	6	7		
	PR	23	17 ± 8		1	16	14		
	SD	7	14 ± 13		0	6	3		
	PD	107	1 ± 1	<0.0001	94	10	6	<0.0001	0.7609
HSCT	No	103	3 ± 2						
	Autologous	64	44 ± 6						
	Allogeneic	87	46 ± 5	<0.0001					

## Data Availability

The data presented in this study are available on request from the corresponding author. The data are not publicly available due to ethical reasons.

## Supplementary Materials

The following are available online at https://www.mdpi.com/article/10.3390/cancers13092075/s1. The supplementary data file (rr NHL in children supplementary data) provides additional information on materials and methods, results, nine tables and six figures. Table S1: First line NHL treatment of the 639 evaluable patients and number of patients who received rituximab as part of first line treatment, Table S2: Characteristics of the 22 patients with r/r NHL alive without HSCT, Table S3: Detailed comparison of the patient and disease characteristics of r/r NHL patients treated without HSCT, with autologous HSCT and with allogeneic HSCT, Table S4: First line Burkitt lymphoma/leukemia treatment of the 254 evaluable patients and number of patients who received rituximab as part of first line treatment, Table S5: Initiated 2nd line treatment in r/r Burkitt lymphoma/leukemia patients, Table S6: Association of patient’s characteristics and response parameters with outcome in r/r Burkitt patients according to type of HSCT, Table S7: Recently published data on refractory and relapsed NHL in children and adolescents, Table S8: Summary of treatment recommendations for the histological subtypes of refractory and relapsed NHL. The recommendations apply to typical cases of r/r NHL. Rare cases with low-risk relapse e. g. very late relapse or relapse after very limited 1st line therapy are discussed in the text, Table S9: Clinical trials for pediatric or adolescent patients with refractory or relapsed NHL. The following table provides information on those trials that are open for recruitment and listed on ClinicalTrials.gov (accessed on 4 March 2021). Advanced search terms: Recruiting Studies | Interventional Studies | NHL | Child; date 2021, April 9th, Figure S1: Reported patients (pts) of refractory and relapsed Non-Hodgkin lymphoma (r/r NHL) and selection of evaluable cases, Figure S2: Probability of survival at 8 years for all r/r NHL (2a), according to response to first-line treatment (2b), according to HSCT status (2c), according to period of NHL diagnosis (2d), according to interval to relapse (2e) and according to remission status after 2nd line treatment (2f), Figure S3: Cumulative incidence of lymphoma related death (CI DOD) and treatment related mortality (CI TRM) for all r/r NHL according to HSCT status (3a), cumulative incidence of lymphoma related death (CI DOD) and treatment related mortality (CI TRM) for r/r NHL with allogenic hematopoietic stem cell transplantation (HSCT) from a matched family donor (3b), cumulative incidence of lymphoma related death (CI DOD) and treatment related mortality (CI TRM) for r/r NHL with allogenic hematopoietic stem cell transplantation (HSCT) from a matched unrelated donor (3c), cumulative incidence of lymphoma related death (CI DOD) and treatment related mortality (CI TRM) for r/r NHL with allogenic hematopoietic stem cell transplantation (HSCT) from a mismatch donor or haploidentical HSCT (3d), Figure S4: Probability of survival at 8 years for T-cell lymphoblastic lymphoma (T-LBL) according to local involvement at relapse (4a), according to time to relapse (4b), according to age at initial diagnosis (4c), according to stage of disease at initial diagnosis (4d), and according to CNS involvement at relapse (4e), Figure S5: Probability of survival at 8 years for diffuse large B-cell lymphoma (DLBCL) according to response to 1st line treatment (5a) and according to time to relapse (5b), Figure S6: Probability of survival at 8 years for r/r Burkitt lymphoma and leukemia with (R)ICE reinduction treatment according to HSCT status (6a) and probability of survival at 8 years for r/r Burkitt lymphoma and leukemia with RVICI reinduction treatment according to HSCT status (6b), cumulative incidence of lymphoma related death (CI DOD) and treatment related mortality (CI TRM) for r/r BL/B-AL with autologous hematopoietic stem cell transplantation (HSCT) (6c), cumulative incidence of lymphoma related death (CI DOD) and treatment related mortality (CI TRM) for r/r BL/B-AL with allogeneic hematopoietic stem cell transplantation (HSCT) (6d), probability of survival at 8 years for r/r BL/B-AL according to initial CNS status (6e), according to response to 1st line treatment (6f), and according to time to r/r BL/B-AL (6g).
