# Supplementary material for: Treatment and Outcome Analysis of 639 Relapsed Non-Hodgkin Lymphomas in Children and Adolescents and Resulting Treatment Recommendations
Source: Cancers (Basel). 2021 Apr 25;13(9):2075. doi: 10.3390/cancers13092075 (PMC8123268; doi:10.3390/cancers13092075)
Supplement: Supplementary file 1 [file cancers-13-02075-s001.zip › cancers-1153263-supplementary.pdf]

cancers

MDPI

Article

Supplementary Materials: Treatment and Outcome Analysis of 639 Relapsed Non-Hodgkin Lymphomas in Children and Adolescents and Resulting Treatment Recommendations

Birgit Burkhardt, Mary Taj, Nathalie Garnier, Veronique Minard-Colin, Volkan Hazar, Karin Mellgren, Tomoo Osumi, Alina Fedorova, Natalia Myakova, Jaime Verdu-Amoros, Mara Andres, Edita Kabickova, Andishe Attarbaschi, Alan Kwok Shing Chiang, Eva Bubanska, Svetlana Donska, Lisa Lyngsie Hjalgrim, Jacek Wachowiak, Anna Pieczonka, Anne Uyttebroeck, Jelena Lazic, Jan Loeffen, Jochen Buechner, Felix Niggli, Monika Csoka, Gergely Krivan, Julia Palma, Amos Burke, Auke Beishuizen, Kristin Koeppen, Stephanie Mueller, Heidi Herbrueggen, Wilhelm Woessmann, Martin Zimmermann, Adriana Balduzzi and Marta Pillon

Supplementary Materials and Methods

The retrospective analysis included eligible cases diagnosed and treated in the year 2000 or later. Data were submitted either on paper CRF or electronically. Eligibility criteria for cases were i) NHL diagnosis, any subtype except for ALCL in order not to interfere with ongoing studies on relapsed ALCL, ii) age at initial NHL diagnosis below 18 years, iii) diagnosis of NHL in 2000 or later to ensure comparable supportive care standards and within 2017, in order to allow a 2-year minimum potential follow-up and iv) refractory disease, disease progression or relapse of NHL. Due to the lack of pieces of information in individual patients, the percentages in the following manuscript always refer to the cohort in which the respective information is available.

The following definitions for refractory disease were applied: in B-NHL, PMLBL, PTCL and patients with B-NHL treatment, refractory CNS disease was diagnosed in cases with persistence of blasts in the cerebrospinal fluid (CSF) prior to the third course of treatment. Refractory bone marrow (BM) disease was defined as persistent BM blasts in BM aspirates prior to the third course and for all other manifestations refractory disease was defined as no reduction in lymphoma size prior to the third course or vital lymphoma cells after the fourth course of treatment. Definition of refractory disease in LBL and patients with ALL-type treatment included cases with less than 35% volume regression at the end of four drug induction, and/or persistence of more than 5% blasts in the BM and/or persistence of blasts in the CSF at the end of a four drug induction, e.g. day 33 in NHL-BFM-like protocol. The term progression was used in cases that responded to treatment but presented with disease progression prior to complete remission. The term relapse was used in cases that achieved CR and presented with disease reoccurrence later on. Because of the limited relevance for the analysis to differentiate progressive disease from relapse and the clinical challenges in defining CR in NHL, the two terms were used synonymously in this analysis. Local relapse was defined as reappearance of NHL at the site that was involved at initial diagnosis. As radiologic residues after the end of treatment are quite frequent in NHL and not necessarily require treatment intensification, all these cases not fulfilling the definition of refractory disease (see above) were excluded from the analysis.

A total of 783 cases from 25 participating countries of the I-BFM and/or EICNHL co-operation were submitted to the data center in Muenster, Germany. Initial diagnosis of NHL was between January 2000 and July 2017. Central review and data clarification led to the exclusion of 144 cases as detailed in Supplementary Figure S1. A total of 639 cases were included in the analysis contributed by Germany (170), Italy (105), France (60), Turkey (42), United Kingdom (41), Sweden and Finland (30), Belarus (26), Japan (20), Spain (19), Hong Kong (18), Czech Republic (17), Moscow (13), Switzerland (13), Austria (10), Slovakia (10), Denmark (10), Poland (7), Ukraine (6), Belgium (6), Serbia (5), Norway (5), Netherlands (4) and Hungary (2). The median follow-up for patients alive is 5.7 years after diagnosis of relapse (0.1-16.4) including eight patients with less than one year follow-up after r/r NHL

Supplementary results

The median interval from initial diagnosis to resistant disease or relapse was 6.2 months, ranging from 0.1 months to 126.2 months. Survival at 8 years for the whole cohort was 34 ± 2% (p 0.001, Supplementary Figure S2a). Survival for patients with refractory diseases according to the above-mentioned strict criteria (n = 43, 7%) was 30 ± 7%. Eighteen

percent of the patients were diagnosed with disease progression and had an OS of 28±4%. Seventy-five percent of the patients suffered relapses with an OS of 36 ± 2% (p 0.001, Supplementary Figure S2b).

After reinduction treatment, 39% of patients underwent allogeneic HSCT resulting in an OS of 47 ± 3%. Twenty-three percent underwent autologous HSCT with an OS of 55±5% and 37% did not undergo HSCT resulting in an OS of 8 ± 2% (Supplementary Figure S2c). The reasons, why patients did not receive HSCT were treatment-related mortality (TRM) during reinduction treatment in 2%, while 32% of patients presented with unmanageable disease progression prior to transplant preventing HSCT and in 4% of r/r NHL HSCT was not planned. The characteristics of the 22 r/r NHL alive without HSCT are detailed in Supplementary Table S1.

Comparisons of the patient's characteristics of the three patient cohorts i) no HSCT, ii) autologous HSCT or iii) allogeneic HSCT revealed significant differences. Among the allogeneic HSCT group, there was a higher proportion of patients diagnosed between 2008 and 2016 compared to the earlier time period, a higher proportion of patients 10 years or younger at diagnosis, more advanced disease stages, especially stage IV disease at initial diagnosis and more frequent BM involvement either at initial diagnosis or at relapse (Supplementary Table S2).

Variables associated with outcome

The outcome of patients diagnosed between 2000 and 2007 was identical to the survival of patients diagnosed between 2008 and 2016 (Supplementary Figure S2d). OS was inferior for patients with advanced stages (p 0.0003) while sex and age were not significantly associated with survival. The probability of survival increased according to the time elapsing between initial diagnosis and the diagnosis of r/r NHL ($p < 0.0001$) (Supplementary Figure S2e). Involved sites, especially BM and CNS involvement at initial diagnosis as well as lymphoma manifestations at the time of relapse were of prognostic relevance (Table 1). The response to second-line treatment and the resulting remission status prior to HSCT showed strong predictive impact on survival. The survival was 61 ± 3% for those 294 patients who achieved 2nd remission prior to HSCT, while survival was intermediate for 29 patients with unconfirmed CR (Cru) or very good partial remission (VGPR) (44 ± 12%), partial remission (n = 54, 34 ± 7%) or stable disease (n = 18, 27 ± 11%) and fatal for those 238 patients who presented with further disease progression during reinduction treatment with OS of 1% (Supplementary Figure S2f, $p < 0.0001$).

Autologous and allogeneic HSCT

For the 150 patients who received autologous HSCT, the cumulative incidence of lymphoma related death (death of disease, CI DOD) was 33 ± 4% compared with the cumulative incidence of treatment related death (CI TRM) of 7 ± 2%. For the 251 patients who underwent allogeneic HSCT, CI DOD was 34 ± 3% and CI TRM 18 ± 2% (Supplementary Figure S3a). The CI DOD and CI TRM in 92 cases transplanted from matched sibling donors were 41 ± 5% and 10 ± 3% compared with CI DOD of 26 ± 4% and CI TRM of 26 ± 4% in 114 patients with matched unrelated donors and 45 ± 8% and 11 ± 5% in the 45 cases with mismatched donors, haploidentical or other donors (Supplementary Figure S3b–d). More detailed analyses on autologous versus allogeneic HSCT are provided in Supplementary Table S2.

Results in Burkitt Lymphoma/Leukaemia

Of the 89 patients in whom 2nd line treatment was initiated with (R)ICE , three-quarters continued with (R)ICE resulting in survival of one third of them (21 patients). Re-induction was modified or intensified in a quarter of patients (21 patients, 5 alive). Sixty of 89 BL/B-AL patients with (R)ICE re-induction achieved HSCT with complete remission (CR) in 39, unconfirmed CR or very good partial remission (CRu(VGPR) in 5, partial remission in 7, stable disease (SD) in 4 and progressive disease (PG) in 5 patients prior to HSCT. OS for 26 patients with autologous HSCT was 46 ± 10% and 35 ± 8% for 34 patients with allogeneic HSCT (Supplementary Figure S6a).

Of the 89 patients in whom 2nd line treatment was initiated with (R)ICE, 60 patients achieved HSCT with complete remission (CR) in 39, unconfirmed CR or very good partial remission (CRu/VGPR) in 5, partial remission (PR) in 7, stable disease (SD) in 4 and progressive disease (PD) in 5 patients prior to HSCT.

Supplementary Table S1. First line NHL treatment of the 639 evaluable patients and number of patients who received rituximab as part of first line treatment.

type of treatment	no of patients	%	rituximab in 1st line treatment
FAB/LMB-type group A	3	<1%	0
FAB/LMB-type group B	59	9%	1
FAB/LMB-type group C	28	4%	1

FAB/LMB-type group C for CNS+	20	3%	0
NHL-BFM-type risk group R1 (5)	4	<1%	0
NHL-BFM-type risk group R2	47	7%	5
NHL-BFM-type risk group R3	77	12%	11
NHL-BFM-type risk group R4	114	18%	23
NHL-BFM-type risk group R4 CNS+	41	6%	5
DA-R-EPOCH	8	1%	7
CHOP/CHOEP	9	1%	3
EURO-LB02-type, ALL-type treatment	210	33%	0
other	16	3%	5
missing	3	<1%	1
total	639		62

Supplementary Table S2. Characteristics of the 22 patients with r/r NHL alive without HSCT; * with ongoing disease or on relapse treatment.

				**initial diagnosis**				**diagnosis to relapse**							**relapse to last follow up**
no	age at diagnosis	gender	histology	BM	CNS	stage	treatment group	(months)		BM	CNS	local	Re-induction treatment	reason for no HSCT	(months)
476	9.3	ma	Burkitt	yes	yes	4	NHL-BFM-type R4 CNS+	4	relapse	yes	yes	no	prephase, R-ICE, non-response	not achieved	1*
266	10.3	male	Burkitt	no	no	2	FAB/LMB-type group A	73	relapse	no	no	yes	FAB BFM group B, resection	not planned	144
345	14.0	female	Burkitt	no	no	3	FAB/LMB-type group C	7	relapse	no	no	yes	R-ICE, rituximab, irradiation	not planned	72
400	9.1	male	Burkitt	no	no	2	FAB/LMB-type group A	4	relapse	no	no	yes	FAB/LMB group C	not planned	55
246	16.2	male	DLBCL	no	no	3	FAB/LMB-type group B	61	relapse	no	no	yes	4x rituximab-GEM-P x2 +radiotherapy 30 Gy	not planned	43
548	17.7	male	DLBCL	no	no	3	NHL-BFM-type R2	32	relapse	no	no	yes	6xR-CHOP, irradiation 30Gy	not planned	44
99	1.2	male	DLBCL	no	no	2	NHL-BFM-type R2	19	relapse	no	no	yes	NHL-BFM courses	not planned	104
596	7.6	female	DLBCL	no	no	3	NHL-BFM-type R3	14	relapse	no	no	yes	NHL BFM 95, R2	not planned	177
350	14.3	female	PMLBL	no	no	3	FAB/LMB-type group C	12	relapse	no	no	yes	R-CHOP	not planned	52
96	11.9	male	B-NHL nfs	no	no	3	NHL-BFM-type R3	5	refractory disease	no	no	yes	ICE	not planned	185
167	4.3	male	T-LBL	yes	no	4	ALL-type	58	relapse	no	no	yes	ALL-REZ BFM2002	not planned	105
310	8.4	male	T-LBL	no	no	3	ALL-type	67	relapse	no	no	yes	NHL-BFM courses	not planned	74
149	11.4	male	T-LBL	no	no	3	ALL-type	40	relapse	no	no	yes	orchidectomy, 15 Gy irradiation, ALL 2007	not planned	45
384	7.7	male	T-LBL	no	no	3	ALL-type	2	refractory disease	no	no	yes	Nelarabine, Veda, EORO-LB Phase M and irradiation	not planned	59
463	13.0	male	T-LBL	yes	no	4	ALL-type	1	refractory disease	no	no	yes	ALL-BFM 2000 HR	not planned	150
164	3.3	female	pB-LBL	no	no	2	ALL-type	46	relapse	no	yes	no	ALL-REZ BFM2002	not planned	131
20	3.3	male	pB-LBL	no	no	3	ALL-type	37	relapse	no	no	yes	ALL-REZ BFM2002	not planned	40
122	2.7	male	pB-LBL	no	no	3	ALL-type	21	relapse	no	no	yes	NHL-BFM courses	not planned	118
379	4.2	male	pB-LBL	no	no	3	ALL-type	15	relapse	no	no	yes	4x rituximab	not planned	75
388	6.5	female	pB-LBL	yes	no	4	ALL-type	6	relapse	no	no	yes	VANDA, 6 MP, MTX, vinblastin	not planned	24
648	9.1	male	pB-LBL	no	no	2	ALL-type	70	relapse	no	no	yes	ALL-REZ BFM2002	not planned	79
504	16.4	female	other NHL	no	no	1	complete resection	3	relapse	no	no	yes	A4-CC-B4-4x rituximab	not planned	52

Supplementary Table S3. Detailed comparison of the patient and disease characteristics of r/r NHL patients treated without HSCT, with autologous HSCT and with allogeneic HSCT. All data refer to patients for whom the relevant variable was known.

		**No**	**HCST**	**autologous**	**HSCT**	**allogeneic**	**HSCT**	**P value no HSCT vs HSCT (auto/allo) (chi²)**	**P value all 3 co-horts (chi²)**	**P value auto vs allo (chi²)**
		N=238	(%)	N=150	(%)	N=251	(%)			
diagnosis	2000-07	125	53	87	58	112	45			
	2008-16	113	48	63	42	139	55	0.4791	0.0270	0.0092
sex	male	173	73	116	77	175	70			
	female	65	27	34	23	76	30	0.9737	0.2545	0.0983
age	<10y	99	42	57	38	131	52			
	≥10 y	139	58	93	62	120	48	0.1940	0.0094	0.0059
stage	I	3	1	8	5	1	1			
	II	10	4	13	9	11	5			
	III	138	60	99	66	124	51			
	IV	80	35	30	20	108	44	0.6099	<0.0001	<0.0001
initial CNS disease	yes	27	12	12	9	37	15	0.7910	0.1070	0.0378
initial BM disease	yes	74	32	27	18	90	37	0.5635	0.0003	<0.0001
CNS disease at relapse	yes	37	16	30	21	61	24	0.0255	0.0540	0.3794
BM disease at relapse	yes	87	37	20	14	91	36	0.0227	<0.0001	<0.0001
local relapse	yes	207	88	127	87	193	77	0.0203	0.0033	0.025
time to relapse	< 3 mo	39	17	15	10	22	9			
	3- ≤ 6 mo	99	42	63	42	70	29			
	6- ≤ 9 mo	32	14	32	21	40	16			
	≥ 9 mo	65	28	39	26	113	46	0.0011	<0.0001	0.0010
remission status at Tx	CR	26	11	89	60	179	71			
	Cru/VGPR	0	0	13	9	16	6			
	PR	3	1	21	14	30	12			
	SD	0	0	13	9	5	2			
	PD	207	88	11	7	20	8	<0.0001	<0.0001	0.0146

Supplementary Table S4. First line Burkitt lymphoma/leukemia treatment of the 254 evaluable patients and number of patients who received rituximab as part of first line treatment.

**type of treatment**	**no of patients**	**%**	**rituximab in 1st line treatment**
FAB/LMB-type group A (1)	3	1%	0
FAB/LMB-type group B	38	15%	1
FAB/LMB-type group C	18	7%	1
FAB/LMB-type group C for CNS+	18	7%	0
NHL-BFM-type risk group R1 (5)	2	1%	0
NHL-BFM-type risk group R2	23	9%	3
NHL-BFM-type risk group R3	34	13%	4
NHL-BFM-type risk group R4	85	33%	16
NHL-BFM-type risk group R4 CNS+	31	12%	4
other	2	1%	1
total	254		30

Supplementary Table S5. Initiated 2nd line treatment in r/r Burkitt lymphoma/leukemia patients. All data refer to cases with respective information available. First line treatment according to FAB group A and NHL-BFM R1 is classified as low risk, FAB group B and NHL-BFM R2 and R3 as intermediate risk and FAB group C and NHL-BFM R4 as high risk.

**initiated 2nd line**	**total**	**intensity of 1st line**	**alive**	**HSCT in pts alive**
NHL-BFM or FAB courses for advanced B-NHL	94	5 low risk<br>48 intermediate risk<br>39 high risk<br>2 other	28	2 without HSCT<br>15 auto HSCT<br>11 allo HSCT
RICE and ICE	89<br>(82+7)	0 low risk<br>31 intermediate risk<br>58 high risk	26	2 without HSCT<br>12 auto HSCT<br>12 allo HSCT
VICI and variants	33	0 low risk<br>10 intermediate risk<br>23 high risk	14	1 auto HSCT<br>13 allo HSCT
DA-R-EPOCH	4		0	
other regimen	34	0 low risk<br>4 intermediate risk<br>30 high risk	5	1 auto HSCT<br>4 allo HSCT

Supplementary Table S6. Association of patient's characteristics and response parameters with outcome in r/r Burkitt patients according to type of HSCT. All data refer to cases with respective information available.

	autologous HSCT			allogeneic HSCT		
	n	OS 8y (±SE) (%)	P value (log-rank)	n	OS 8y (±SE) (%)	p value (log-rank)
sex						
male	59	46±7		69	43±6	
female	5	20±18	0.11	18	56±12	0.42
age						
< 10 years	30	50±9		54	50±7	
≥ 10 years	34	39±9	0.81	33	39±9	0.36
period of diagnosis						
2000-07	41	36±8		38	47±8	
2008-16	23	57±12	0.045	49	45±7	0.75
initial stage of disease						
I	1	100		0		
II	4	50±25		4	100	
III	38	47±9		27	52±10	
IV	20	30±10	0.25	56	39±7	0.18
BM involvement at initial diagnosis						

yes	19	26±10		42	36±7	
no	45	51±8	0.41	35	60±8	0.13
BM involvement at relapse						
yes	13	38±13		37	51±8	
no	50	44±7	0.57	50	42±7	0.30
CNS at diagnosis						
yes	7	14±13		20	25±10	
no	57	48±7	0.066	67	52±6	0.021
CNS at relapse						
yes	17	32±12		30	37±9	
no	46	47±7	0.25	57	51±7	0.26
local relapse						
yes	50	43±7		65	49±6	
no	13	45±14	0.88	22	36±10	0.43
response to 1st line						
refractory	4	25±22		2	0	
progression	10	30±14		13	31±13	
relapse	50	48±7	0.26	72	50±6	0.0092
interval to r/r disease						
<3 months	7	43±19		10	30±14	
3–6 months	40	37±8		39	38±8	
6–9 months	12	54±15		20	50±11	
≥ 9 months	5	80±18	0.23	15	66±12	0.13
response to 2nd line						
2nd CR	39	58±8		61	57±6	
CRu or VGPR	4	75±22		6	33±19	
PR	10	20±13		12	17±11	
SD	4	0		3	33±27	
progression	6	17±15	0.0076	5	0	< 0.0001

Supplementary Table S7. Recently published data on refractory and relapsed NHL in children and adolescents.

Reference	NHL subtype	No of pts	No of pts with SCT	OS
**Prospective trials**				
Burke 2020	B-NHL	21	7	EFS 6/21
Griffin 2009	B-NHL	20	6	38%
**Retrospective series**				
Woessmann 2020*	Burkitt	75	46	27%
Rigaud 2019	B-NHL	33	21	36%
Fujita 2019	B-NHL	79	48 allogeneic<br>31 autologous	32% allogeneic<br>55% autologous
Naik 2019	DLBCL	8	8	63%
Cairo 2018	B-NHL	104	n.a.	22%
Osumi 2016	B-NHL	33	23	49%
Jourdain 2015	B-NHL	67	41	30%
Kim 2014	B-NHL			
Anoop 2012	B-NHL	33	16	27%
Gross 2010	Burkitt	41	24 allogeneic<br>17 autologous	31% allogeneic<br>27% autologous
Gross 2010	DLBCL	52	17 allogeneic<br>35 autologous	50% allogeneic<br>52% autologous
Naik 2019	LBL	9	9	17%
Michaux 2016	LBL	23	10	8%

Gross 2010	LBL	53	39 allogeneic<br>14 autologous	40% allogeneic<br>4% autologous
Mitsui 2009	LBL	48	33	43±7%
Burkhardt 2009	LBL	34	13	14±6

Including preferentially reports and patients treated after the year 2000. * The publication reports 157 Burkitt Lymphoma/Leukaemia patients diagnosed between 1986 and 2016. The 3- year OS was reported to be 18% for the whole cohort. For more detailed analyses the publication focused on 75 patients treated in the year 2000 or later.

Supplementary Table S8. Summary of treatment recommendations for the histological subtypes of refractory and relapsed NHL. The recommendations apply to typical cases of r/r NHL. Rare cases with low-risk relapse e. g. very late relapse or relapse after very limited 1st line therapy are discussed in the text.

General receommendations	NHL subtype	2nd line treatment	HSCT	Conditioning regimen
• maintain high time- and dose-intense treatment<br>• avoid treatment delays<br>• consider treatment continuation prior to full hematological recovery<br>• perform systemic treatment (limited/no role for irradiation and surgery)<br>• achieve CR prior to HSCT<br>• early taper of immunosuppression after allogeneic HSCT	Lymphoblastic lymphoma	intense treatment courses analogue to high-risk ALL or relapsed ALL protocols	allogeneic HSCT	TBI based conditioning at least for T-LBL
	Diffuse large B-cell lymphoma	2-3 courses R-ICE	autologous HSCT	BEAM or busulfan based regimen
	Burkitt lymphoma and leukemia	2-3 courses R-ICE or R-VICI	autologous or allogeneic HSCT	BEAM or busulfan based regimen for autologous HSCT<br>TBI based, Burkitt-specific RIC or busulfan based regimen for allogeneic HSCT

Supplementary Table S9. Clinical trials for pediatric or adolescent patients with refractory or relapsed NHL. The following table provides information on those trials that are open for recruitment and listed on ClinicalTrials.gov. Advanced search terms: Recruiting Studies | Interventional Studies | NHL | Child; date 2021, April 9th.

No	Official title	ClinicalTrials.gov Identifier
	**CAR T cell trials**	
1	Phase 1/2 Dose Escalation and Preliminary Efficacy of CD19 Directed Car T Cells Generated Using The Miltenyi Clinimacs Prodigy System (UCD19 CarT) in Pediatric Patients With Relapsed and/or Refractory B-Cell Acute Lymphoblastic Leukemia (B-ALL) and B-Cell Non-Hodgkins Lymphoma(B-NHL)	NCT04544592
2	Treatment of Decitabine-primed Tandem Targeting CD19 and CD20 Chimeric Antigen Receptor T Cells Plus Epigenetic Agents in Aggressive Relapsed and/or Refractory Non-Hodgkin's Lymphoma Patients With Huge Tumor Burden	NCT04553393
3	A Phase 1/2, Open-label, Single Arm, Multicohort, Multicenter Trial to Evaluate the Safety and Efficacy of JCAR017 in Pediatric Subjects With Relapsed/Refractory (r/r) B-cell Acute Lymphoblastic Leukemia (B-ALL) and B-cell Non-Hodgkin Lymphoma (B-NHL).	NCT03743246
4	Adoptive Immunotherapy for Children CD19+ Leukemia and Non-Hodgkin Lymphoma With CD19-TriCAR-T/SILK Cell	NCT03910842
5	A Phase 1/2 Multi-Center Study Evaluating the Safety and Efficacy of KTE-X19 in Pediatric and Adolescent Subjects With Relapsed/Refractory B-precursor Acute Lymphoblastic Leukemia or Relapsed/Refractory B-Cell Non-Hodgkin Lymphoma (ZUMA-4)	NCT02625480
6	Phase I/ II Study of Cluster of Differentiation 19 (CD19) Specific CAR-T Cells (ISIKOK 19) in Relapsed/Refractory Acute Lymphoblastic Leukemia (ALL) and Non Hodgkin Lymphoma (NHL)	NCT04206943
7	A Multicenter Clinical Study on the Safety and Efficacy of CAR-T in the Treatment of Relapsed / Refractory Non Hodgkin's Lymphoma	NCT04666168
8	Clinical Trial of SL1904B CAR-T Cells for Relapsed or Refractory Non-Hodgkin Lymphoma	NCT04447547
9	Safety and Efficacy of Chimeric Antigen Receptor T Cell (CAR-T) Treating Relaspe/Refractory CD19/CD20/CD22/CD30 Postive Non-Hodgkin Lymphoma	NCT03196830

10	Clinical Trial for the Safety and Efficacy of Humanized CD19 CAR-T Cells Therapy for Patients With Relapsed and/or Refractory B-cell Acute Lymphoblastic Leukemia and B-cell Non-Hodgkin's Lymphoma	NCT04532268
11	Phase I/II Study of Anti-CD19 Chimeric Antigen Receptor-Expressing T Cells in Pediatric Patients Affected by Relapsed/Refractory CD19+ Acute Lymphoblastic Leukemia and Non Hodgkin Lymphoma	NCT03373071
12	Decitabine-primed Tandem Targeting CD19 and CD20 Chimeric Antigen Receptor T Cells Treatment in Relapsed and/or Refractory Non-Hodgkin's Lymphoma Patients	NCT04697940
13	Phase I Study of Activated T-Cells Expressing Second or Third Generation CD19-Specific Chimeric Antigen Receptors for Advanced B-Cell Non-Hodgkin's Lymphoma, Acute Lymphocytic Leukemia and Chronic Lymphocytic Leukemia (SAGAN)	NCT01853631
14	Immunotherapy for High Risk/Relapsed CD19+ Acute Lymphoblastic Leukaemia, B-cell Non-Hodgkin's Lymphoma (B-NHL) and Chronic Lymphocytic Leukaemia (CLL)/ Small Lymphocytic Lymphoma (SLL) Using CAR T-cells to Target CD19	NCT02935257
15	Clinical Trial for the Safety and Efficacy of CAR-T Cells Therapy for Patients With the Central Nervous System Involvement of Relapsed and/or Refractory B-cell Acute Lymphoblastic Leukemia or B-cell Non-Hodgkin's Lymphoma	NCT04532203
16	Phase Ib/II Study of the Administration of T Lymphocytes Expressing the CD30 Chimeric Antigen Receptor (CAR) for Relapsed/Refractory CD30+ Hodgkin's Lymphoma and CD30+ Non-Hodgkin's Lymphoma	NCT02690545
17	Clinical Trial for the Safety and Efficacy of Murine CD19 CAR-T Cells Therapy for Patients With Relapsed and/or Refractory B-cell Acute Lymphoblastic Leukemia and B-cell Non-Hodgkin's Lymphoma	NCT04532281
18	A Safety and Efficacy Study of CD19-UCART (Allogeneic Engineered T-cells Expressing Anti-CD19 Chimeric Antigen Receptor) in Patients With Relapsed or Refractory B-cell Hematologic Malignancies	NCT03229876
19	Phase I Dose Escalation Study of Anti-CD22 Chimeric Receptor T Cells in Pediatric and Young Adults With Recurrent or Refractory CD22-expressing B Cell Malignancies	NCT02315612
20	A Phase 1 / 2 Single Arm Study of T-cells Expressing Anti-CD19 Chimeric Antigen Receptor in Pediatric and Young Adult Patients With B-cell Malignancies	NCT02772198
21	A Study of CTA101 UCAR-T Cell Injection in Patients With Relapsed or Refractory CD19+ B-line Hematological Malignancy	NCT04227015
22	A Study of CT-RD06 Cell Injection in Patients With Relapsed or Refractory CD19+ B-cell Hematological Malignancy	NCT04226989
23	Phase I Study of Relapsed CD30 Expressing Lymphoma Treated With CD30 CAR T Cells (RELY-30)	NCT02917083
24	CD30-directed Chimeric Antigen Receptor T (CART30) Therapy in Relapsed and Refractory CD30 Positive Lymphomas	NCT02259556
25	Anti-CD5 CAR T Cells for Relapsed/Refractory T Cell Malignancies	NCT04594135
26	Phase 1 Therapy With Manufactured Autologous T-Cells Expressing a Second Generation Chimeric Antigen Receptor (CAR) for Treatment of T-Cell Malignancies Expressing CD5 Antigen	NCT03081910
27	Phase I Study of Activated T Lymphocytes Expressing Chimeric Antigen Receptors for Therapy of Relapsed CD19-Positive Malignancies Post-Allogeneic Hematopoietic Stem Cell Transplantation Infused Only After Engraftment (CARPASCIO)	NCT02050347
28	A Phase 1b/2 Multi-center, De-centralized, Dose Selection Study of Autologous CD19-directed Chimeric Antigen Receptor (CAR) T-cells in Patients With Relapsed/Refractory Aggressive Lymphoma or Acute Lymphoblastic Leukemia (ALL)	NCT03938987
29	Adoptive Immunotherapy for Hematological Malignancy With Novel CAR-T Cells.	NCT04191941
30	A Phase 1 Study Evaluating the Safety and Activity of Allogeneic CD30 Chimeric Antigen Receptor Epstein-Barr Virus-Specific T Lymphocytes (CD30.CAR-EBVSTs) in Patients With Relapsed or Refractory CD30-Positive Lymphomas	NCT04288726
	**Trials with antibodies or antibody-drug conjugates**	
31	Chemoimmunotherapy With Obinutuzumab, Ifosfamide, Carboplatin and Etoposide (O-ICE) in Children, Adolescents and Young Adults With Recurrent Refractory CD20+ Mature B-NHL	NCT02393157
32	A Phase I/Ib Open-label, Multi-center Dose Escalation Study of JBH492 in Patients With Relapsed/Refractory Chronic Lymphocytic Leukemia (CLL) and Non-Hodgkin's Lymphoma (NHL)	NCT04240704

33	Bispecific Antibody AFM13 Combined With NK Cells for Patients With Recurrent or Refractory CD30 Positive Hodgkin or Non-Hodgkin Lymphomas	NCT04074746
	**Trials testing other drugs (including the NCI-COG basket trial)**	
34	A Phase I Trial of Pevonedistat in Combination With Induction Chemotherapy for Adolescent and Young Adults With Relapsed/Refractory Acute Lymphoblastic Leukemia or Lymphoblastic Non-Hodgkin Lymphoma	NCT03349281
35	Phase 1, First-in-human, Open-label Dose Escalation and Cohort Expansion Study of KB-0742 in Patients With Relapsed or Refractory Solid Tumors or Non-Hodgkin Lymphoma	NCT04718675
36	NCI-COG Pediatric MATCH (Molecular Analysis for Therapy Choice) - Phase 2 Subprotocol of JNJ-42756493 (Erdafitinib) in Patients With Tumors Harboring FGFR1/2/3/4 Alterations	NCT03210714
37	NCI-COG Pediatric MATCH (Molecular Analysis for Therapy Choice) Screening Protocol	NCT03155620
38	NCI-COG Pediatric MATCH (Molecular Analysis For Therapy Choice)- Phase 2 Subprotocol of LY3023414 in Patients With Solid Tumors	NCT03213678
39	NCI-COG Pediatric MATCH (Molecular Analysis for Therapy Choice)- Phase 2 Subprotocol of Vemurafenib in Patients With Tumors Harboring BRAF V600 Mutations	NCT03220035
40	NCI-COG Pediatric MATCH (Molecular Analysis for Therapy Choice)- Phase 2 Subprotocol of Olaparib in Patients With Tumors Harboring Defects in DNA Damage Repair Genes	NCT03233204
41	NCI-COG Pediatric MATCH (Molecular Analysis for Therapy Choice) - Phase 2 Subprotocol of LOXO-101 (Larotrectinib) in Patients With Tumors Harboring Actionable NTRK Fusions	NCT03213704
42	NCI-COG Pediatric MATCH (Molecular Analysis for Therapy Choice)- Phase 2 Subprotocol of Ensartinib in Patients With Tumors Harboring ALK or ROS1 Genomic Alterations	NCT03213652
43	NCI-COG Pediatric MATCH (Molecular Analysis for Therapy Choice) - Phase 2 Subprotocol of Palbociclib in Patients With Tumors Harboring Activating Alterations in Cell Cycle Genes	NCT03526250
44	NCI-COG Pediatric MATCH (Molecular Analysis for Therapy Choice) - Phase 2 Subprotocol of LOXO-292 in Patients With Tumors Harboring RET Gene Alterations	NCT04320888
45	NCI-COG Pediatric MATCH (Molecular Analysis for Therapy Choice)- Phase 2 Subprotocol of Tipifarnib in Patients With Tumors Harboring HRAS Genomic Alterations	NCT04284774
46	NCI-COG Pediatric MATCH (Molecular Analysis for Therapy Choice) - Phase 2 Subprotocol of AG-120 (Ivosidenib) in Patients With Tumors Harboring IDH1 Mutations	NCT04195555
47	A Prospective Randomized Controlled Phase II Clinical Trial of Metformin in the Maintenance Therapy of High Risk Diffuse Large B Lymphoma / Stage III Follicular Lymphoma Patients With Complete Remission	NCT03600363
48	Phase 2 Trial of Nivolumab in Epstein-Barr Virus (EBV)-Positive Lymphoproliferative Disorders and EBV-Positive Non-Hodgkin Lymphomas	NCT03258567
49	A Phase 1 Study of the Safety and Pharmacokinetics of Venetoclax in Pediatric and Young Adult Patients With Relapsed or Refractory Malignancies	NCT03236857
50	An Open-Label, Dose Escalation, Efficacy, and Safety Study of CLR 131 in Children, Adolescents, and Young Adults With Select Solid Tumors, Lymphoma, and Malignant Brain Tumors	NCT03478462
	**Trials stem cell transplantation trials**	
51	Autologous Stem Cell Transplant In Patients With Hodgkin Lymphoma (HL) and Non-Hodgkin Lymphomas (NHL)	NCT03125642
52	Gemcitabine/Clofarabine/Busulfan and Allogeneic Transplantation for Aggressive Lymphomas	NCT01701986
53	Safety, Efficacy and Feasibility of Haploidentical Stem Cell Transplantation (Haplo-SCT) Using Post-Transplant Cyclophosphamide (PTCy) as an Alternative Donor Source for Patients Who Lack a Matched Sibling/Unrelated Donor Options	NCT03088709
54	Cord Blood Transplantation in Children and Young Adults With Hematologic Malignancies and Non-malignant Disorders	NCT04644016
55	TCRαβ-depleted Progenitor Cell Graft With Additional Memory T-cell DLI, Plus Selected Use of Blinatumomab, in Naive T-cell Depleted Haploidentical Donor Hematopoietc Cell Transplantation for Hematologic Malignancies	NCT03849651
56	Provision of TCRγδ T Cells and Memory T Cells Plus Selected Use of Blinatumomab in Naïve T-cell Depleted Haploidentical Donor Hematopoietic Cell Transplantation for Hematologic Malignancies Relapsed or Refractory Despite Prior Transplantation	NCT02790515
57	Umbilical Cord Blood Transplantation Using a Myeloablative Preparative Regimen for the Treatment of Hematological Diseases	NCT01962636

58	Transplantation of Umbilical Cord Blood for Patients With Hematological Diseases With Cyclophosphamide/Fludarabine/Total Body Irradiation or Cyclophosphamide/Fludarabine/Thiotepa/Total Body Irradiation Myeloablative Preparative Regimen	NCT00719888
59	Myeloablative Allogeneic Hematopoietic Cell Transplantation Using a Related or Unrelated Donor for the Treatment of Hematological Diseases	NCT03314974
60	A Two Step Approach to Allogeneic Hematopoietic Stem Cell Transplantation From HLA Partially-Matched Related Donors for Patients With Hematologic Malignancies	NCT02566395
61	Allogeneic Hematopoietic Stem Cell Transplantation Using Reduced Intensity Conditioning (RIC) for the Treatment of Hematological Diseases [MT2015-32]	NCT02661035
62	TCR-αβ+ and CD19+ Depleted KIR/KIR Ligand-mismatched Haploidentical Hematopoietic Stem Cell Transplant and Zoledronate for Pediatric Relapsed/Refractory Hematologic Malignancies and High Risk Solid Tumors	NCT02508038
63	Clinical Transplant-Related Long-term Outcomes of Alternative Donor Allogeneic Transplantation (BMT CTN 1702)	NCT03904134
	**Other cellular therapies trials**	
64	Immunotherapy With Ex Vivo-Expanded Cord Blood-Derived NK Cells Combined With Rituximab High-Dose Chemotherapy and Autologous Stem Cell Transplant for B-Cell Non-Hodgkin's Lymphoma	NCT03019640
65	A Study of CTA30X Cell Injection in the Treatment of Relapsed or Refractory CD19-positive B-line Hematological Malignancies	NCT04689204
66	Dose Escalation Study Phase I/II of Umbilical Cord Blood-Derived CAR-Engineered NK Cells in Conjunction With Lymphodepleting Chemotherapy in Patients With Relapsed/Refractory B-Lymphoid Malignancies	NCT03056339
67	Administration of Rapidly Generated EBV-Specific Cytotoxic T-Lymphocytes To Patients With EBV-Positive Lymphoma	NCT01555892
68	Use of T-allo10 Cell Infusions Combined With Mismatched Related or Mismatched Unrelated Hematopoietic Stem Cell Transplantation (HSCT) for Hematologic Malignancies	NCT03198234
69	Allogeneic Natural Killer T-Cells Expressing CD19 Specific Chimeric Antigen Receptor and Interleukin-15 in Relapsed or Refractory B-Cell Malignancies	NCT03774654
70	ADMINISTRATION OF MOST CLOSELY MATCHED THIRD PARTY RAPIDLY GENERATED LMP, BARF1 and EBNA1 SPECIFIC CYTOTOXIC T-LYMPHOCYTES TO PATIENTS WITH EBV-POSITIVE LYMPHOMA AND OTHER EBV-POSITIVE MALIGNANCIES	NCT02287311
71	Cord Blood Ex-Vivo MSC Expansion Plus Fucosylation to Enhance Homing and Engraftment	NCT03096782
72	Personalized NK Cell Therapy in Cord Blood Transplantation	NCT02727803

Suppl. Fig 1

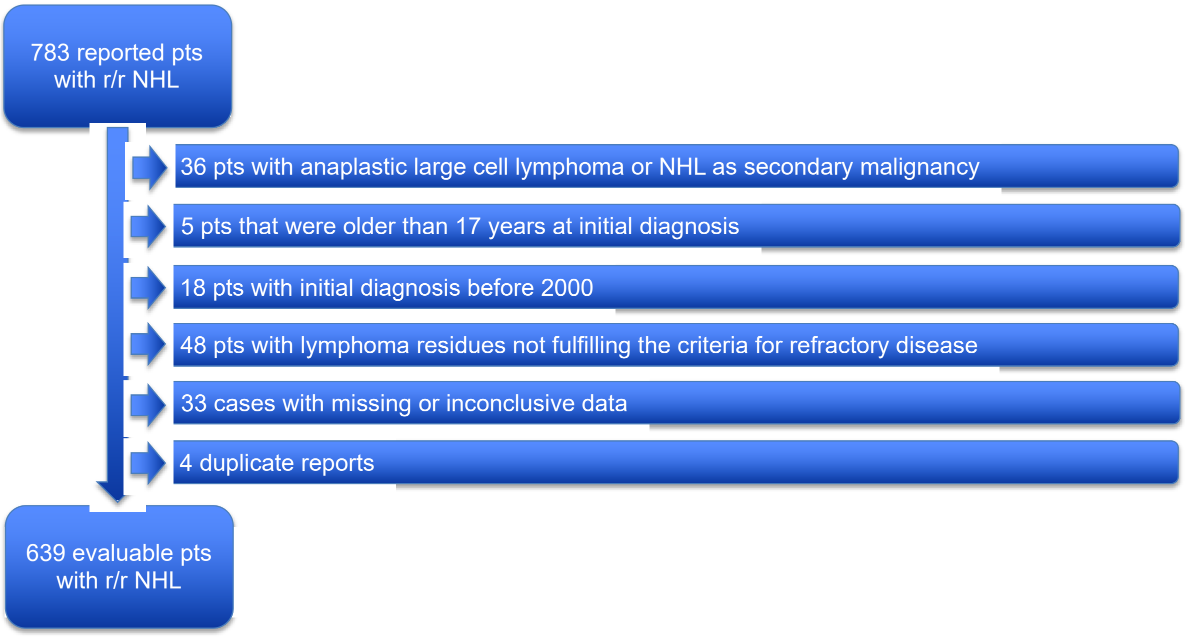

Supplementary Figure S1. Reported patients (pts) of refractory and relapsed Non-Hodgkin lymphoma (r/r NHL) and selection of evaluable cases.

Suppl. Fig 2a Survival in r/r NHL (8 years)

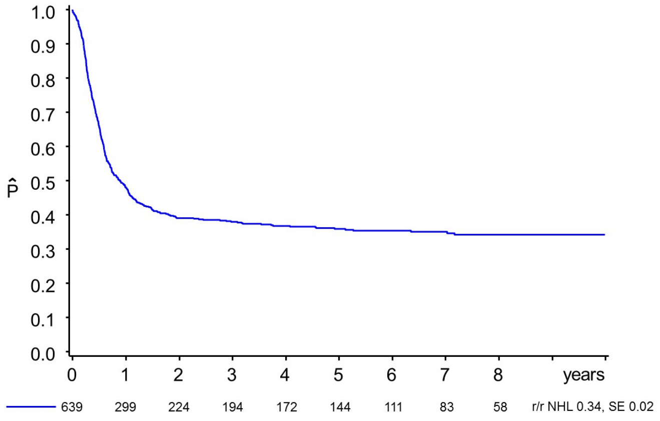

Suppl. Fig 2b Survival in r/r NHL according to response to 1st line treatment (8 years)

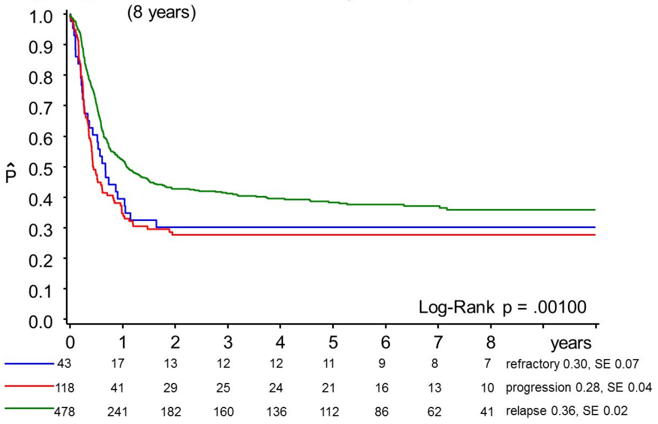

Suppl. Fig 2c Survival in r/r NHL according to HSCT (8 years)

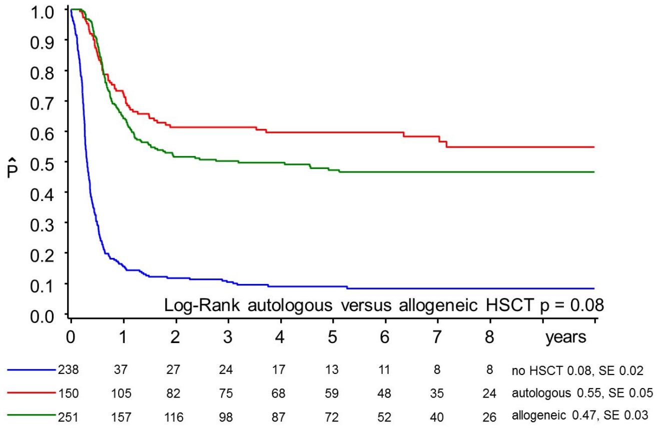

Suppl. Fig 2d Survival in r/r NHL according to period of initial diagnosis (8 years)

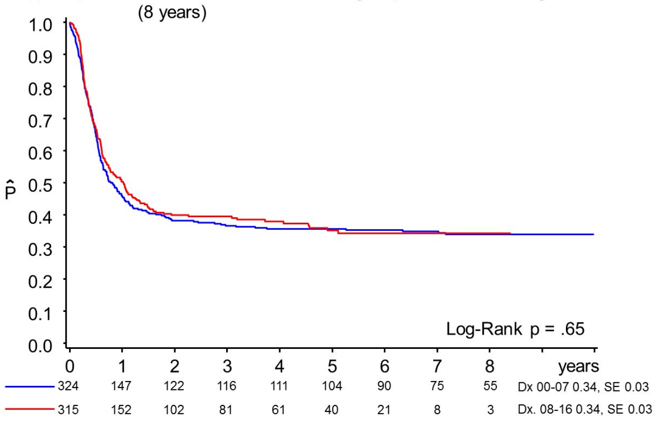

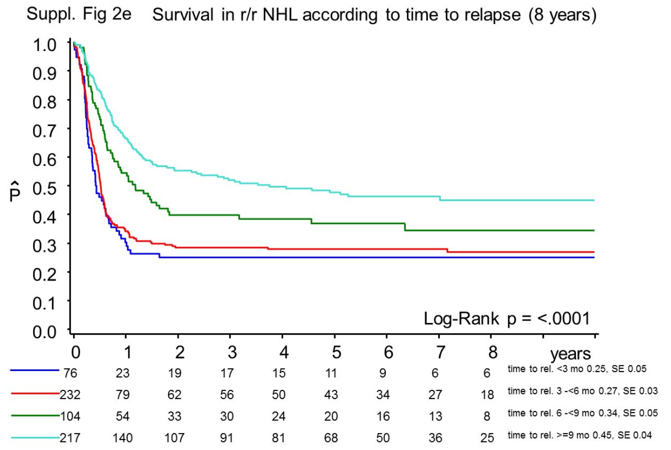

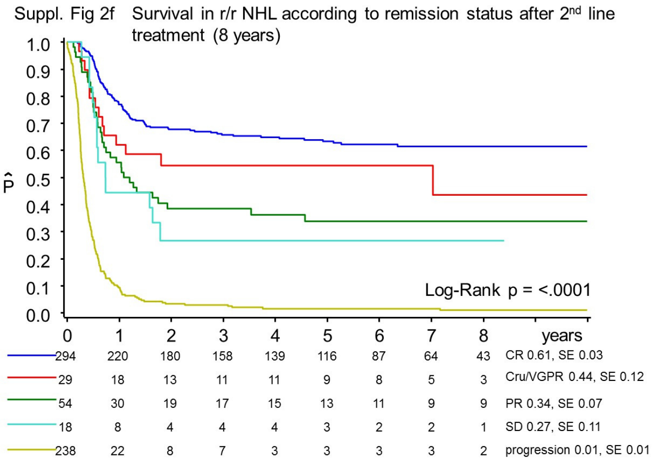

Supplementary Figure S2. Probability of survival at 8 years for all r/r NHL (2a), according to response to 1st line treatment (2b), according to HSCT status (2c), according to period of NHL diagnosis (2d), according to interval to relapse (2e) and according to remission status after 2nd line treatment (2f). r/r NHL: refractory, progressive or relapsed NHL; HSCT: hematopoietic stem cell transplantation; time to rel.: interval between initial diagnosis and relapse; CR: complete remission; Cru: unconfirmed complete remission; VGPR: very good partial remission; PR: partial remission; SD: stable disease.

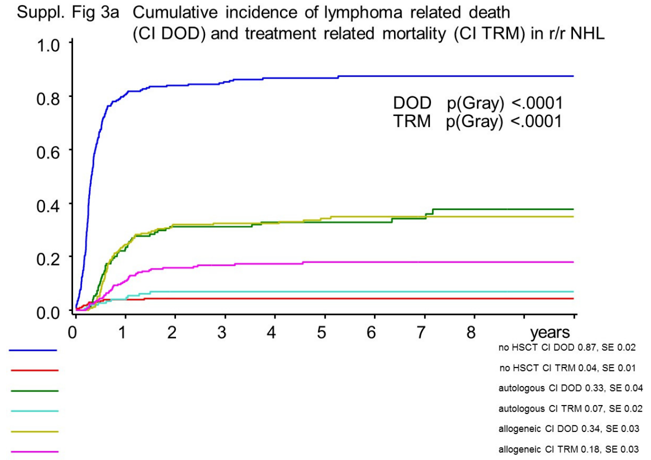

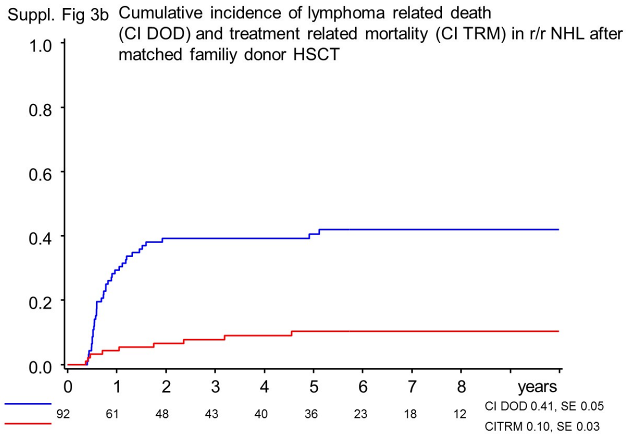

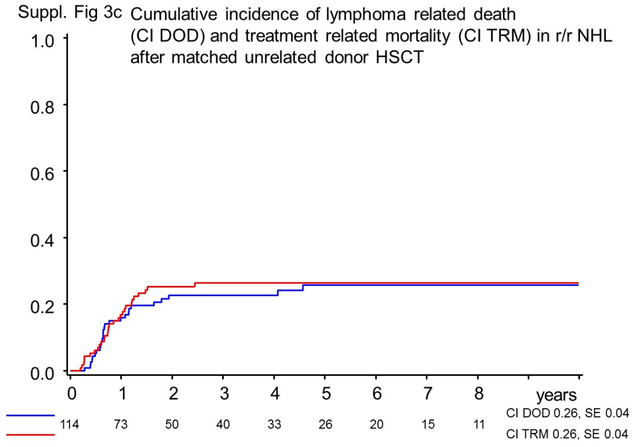

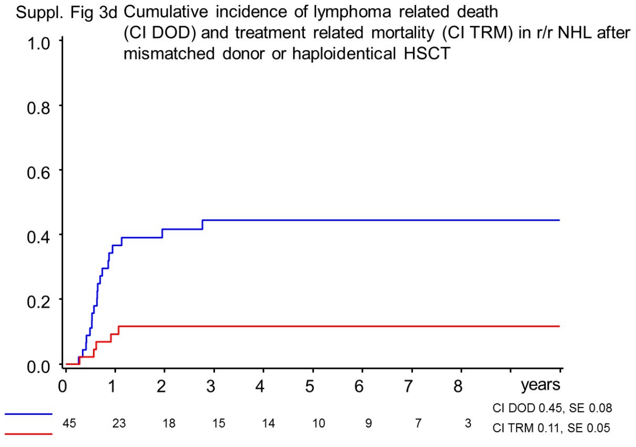

Supplementary Figure S3. Cumulative incidence of lymphoma related death (CI DOD) and treatment related mortality (CI TRM) for all r/r NHL according to HSCT status (3a), cumulative incidence of lymphoma related death (CI DOD) and treatment related mortality (CI TRM) for r/r NHL with allogenic hematopoietic stem cell transplantation (HSCT) from a matched family donor (3b), cumulative incidence of lymphoma related death (CI DOD) and treatment related mortality

(CI TRM) for r/r NHL with allogenic hematopoietic stem cell transplantation (HSCT) from a matched unrelated donor (3c), cumulative incidence of lymphoma related death (CI DOD) and treatment related mortality (CI TRM) for r/r NHL with allogenic hematopoietic stem cell transplantation (HSCT) from a mismatch donor or haploidentical HSCT (3d). HSCT: hematopoietic stem cell transplantation; Dx: NHL diagnosis; mo: months.

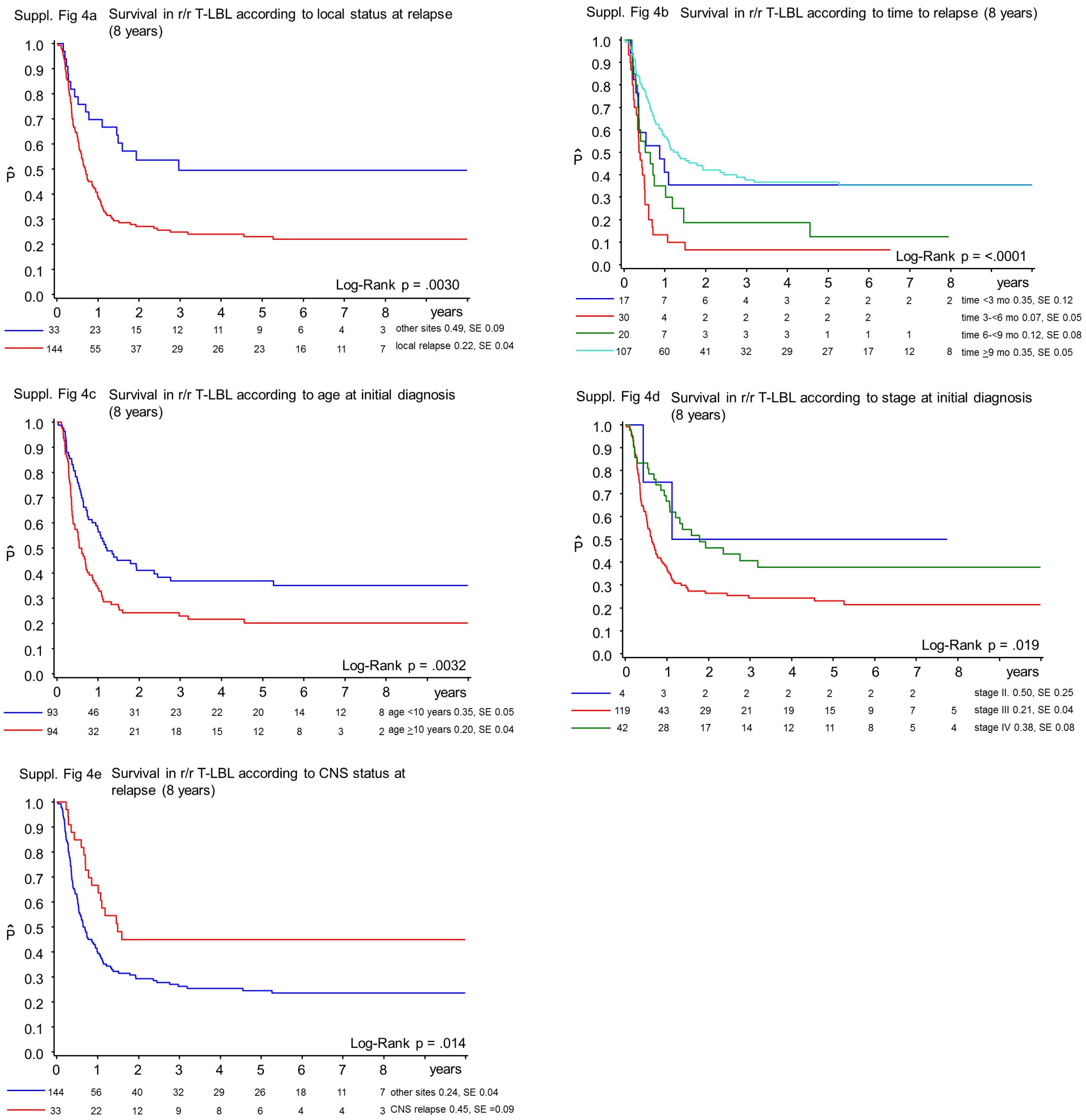

Supplementary Figure S4. Probability of survival at 8 years for T-cell lymphoblastic lymphoma (T-LBL) according to local involvement at relapse (4a), according to time to relapse (4b), according to age at initial diagnosis (4c), according to stage of disease at initial diagnosis (4d), and according to CNS involvement at relapse (4e). HSCT: hematopoietic stem cell transplantation; time to rel.: interval between initial diagnosis and relapse; CNS: central nervous system; mo: months.

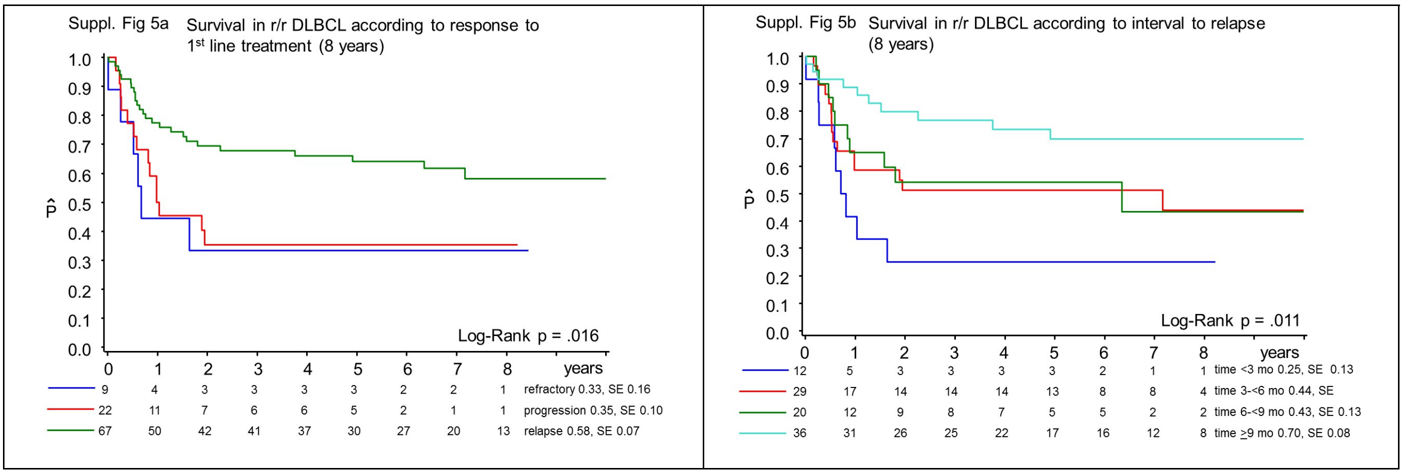

Supplementary Figure S5. Probability of survival at 8 years for diffuse large B-cell lymphoma (DLBCL) according to response to 1st line treatment (5a) and according to time to relapse (5b). HSCT: hematopoietic stem cell transplantation; time to rel.: interval between initial diagnosis and relapse; mo: months.

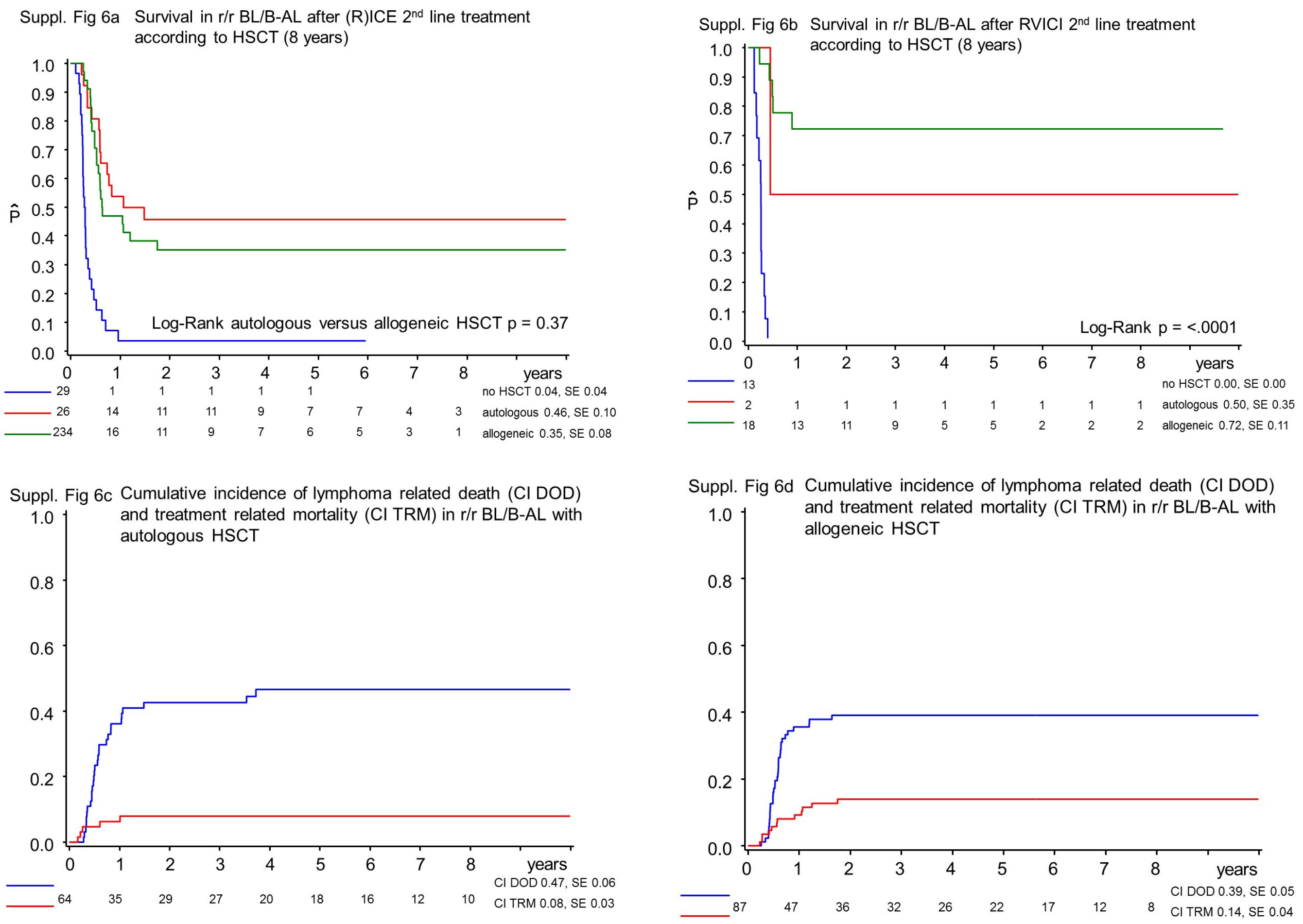

Suppl. Fig 6e Survival in r/r BL/B-AL according to initial CNS status (8 years)

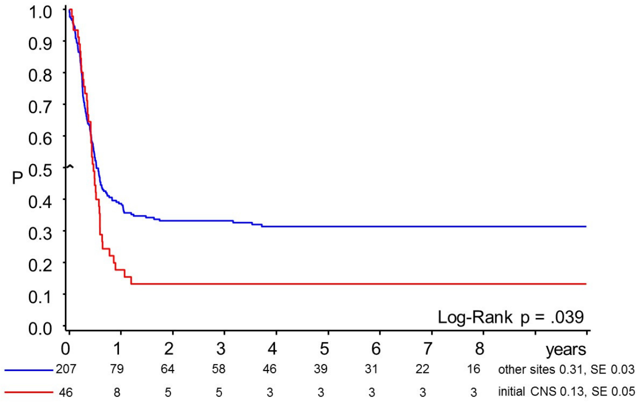

Suppl. Fig 6f Survival in r/r BL/B-AL according to response to 1st line treatment (8 years)

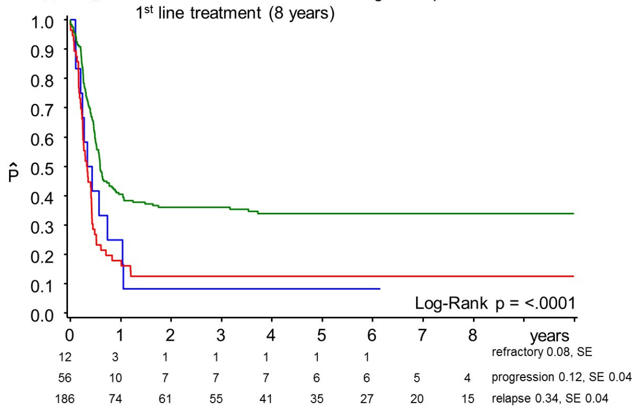

Suppl. Fig 6g Survival in r/r BL/B-AL according to time to relapse (8 years)

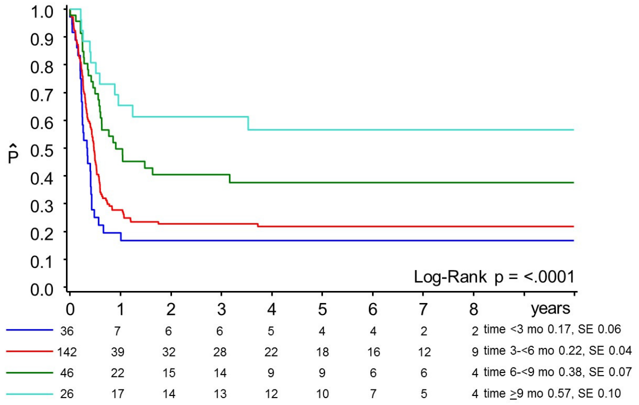

Supplementary Figure S6. Probability of survival at 8 years for r/r Burkitt lymphoma and leukemia with (R)ICE reinduction treatment according to HSCT status (6a) and probability of survival at 8 years for r/r Burkitt lymphoma and leukemia with RVICI reinduction treatment according to HSCT status (6b), cumulative incidence of lymphoma related death (CI DOD) and treatment related mortality (CI TRM) for r/r BL/B-AL with autologous hematopoietic stem cell transplantation (HSCT) (6c), cumulative incidence of lymphoma related death (CI DOD) and treatment related mortality (CI TRM) for r/r BL/B-AL with allogeneic hematopoietic stem cell transplantation (HSCT) (6d), probability of survival at 8 years for r/r BL/B-AL according to initial CNS status (6e), according to response to 1st line treatment (6f), and according to time to r/r BL/B-AL (6g). HSCT: hematopoietic stem cell transplantation; (R)ICE: rituximab plus ifosfamide, carboplatin, etoposide; RVICI: rituximab plus vincristine, ifosfamide, carboplatin, idarubicin, and dexamethasone; CNS: central nervous system; time to rel.: interval between initial diagnosis and relapse; mo: months.
